# Biodegradable Zn-0.8Mg-0.2Sr alloy as an internal fixation material exhibits controlled degradation with enhanced osteogenesis

**DOI:** 10.1039/d5ra02009c

**Published:** 2025-08-22

**Authors:** Yuting Tian, Yichen Xu, Jan Pinc, Jaroslav Fojt, Vojtěch Hybášek, Jiří Kubásek, Šárka Msallamová, Yong Xiang, Min Guo, Jaroslav Čapek, Ping Li, Tao Hu

**Affiliations:** a State Key Laboratory of Oral Diseases & National Center for Stomatology & National Clinical Research Center for Oral Diseases & Frontier Innovation Center for Dental Medicine Plus, West China Hospital of Stomatology, Sichuan University Chengdu 610041 China hutao@scu.edu.cn; b FZU – The Institute of Physics, Czech Academy of Sciences Na Slovance 1999/2 Prague 8 182 00 Czech Republic capekj@fzu.cz; c University of Chemistry and Technology Prague, Faculty of Chemical Technology, Department of Metals and Corrosion Engineering Prague 6 – Dejvice Czech Republic; d SINOPEC Key Laboratory of Research and Application of Medical and Hygienic Materials, SINOPEC Beijing Research Institute of Chemical Industry Co., Ltd. Beijing 100013 China; e Department of Prosthodontics, School and Hospital of Stomatology & Guangdong Engineering Research Center of Oral Restoration and Reconstruction & Guangzhou Key Laboratory of Basic and Applied Research of Oral Regenerative Medicine, Guangzhou Medical University Guangzhou 510180 China pingli@gzhmu.edu.cn

## Abstract

Zinc (Zn) and its alloys are promising candidates for biodegradable metals in medical applications. However, their clinical use in internal fixation is hindered by low mechanical strength, uncontrolled corrosion, and insufficient bioactivity. To address these issues, we developed an extruded Zn-0.8Mg-0.2Sr ternary alloy and systematically evaluated its biological performance. *In vitro* corrosion tests indicated that Zn-0.8Mg-0.2Sr exhibited superior corrosion resistance, attributed to a dense passivation layer that provided effective protection, controlled degradation kinetics, and milder Zn^2+^ release. The cytotoxicity of Zn-0.8Mg-0.2Sr toward pre-osteoblasts was concentration-dependent. Within the non-cytotoxic concentration range (Zn^2+^ ≤8.98 μg mL^−1^), Zn-0.8Mg-0.2Sr promoted osteogenic differentiation more effectively than pure Zn. Further *in vivo* studies confirmed favorable biocompatibility and more uniform degradation of Zn-0.8Mg-0.2Sr, with reduced pitting corrosion and structural collapse. Notably, Zn-0.8Mg-0.2Sr exhibited superior performance in promoting bone regeneration and anti-inflammatory immunomodulation compared to pure Zn. These findings highlight Zn-0.8Mg-0.2Sr as a promising alternative to conventional internal fixation materials, offering favorable biocompatibility, controlled biodegradability, and enhanced osteogenesis.

## Abstract 2

Zn-0.8Mg-0.2Sr ternary alloy facilitates uniform degradation and releases Zn^2+^, Mg^2+^, and Sr^2+^, synergistically promoting anti-inflammatory immunomodulation, bone regeneration, and bone remodeling.

## Graphical abstract

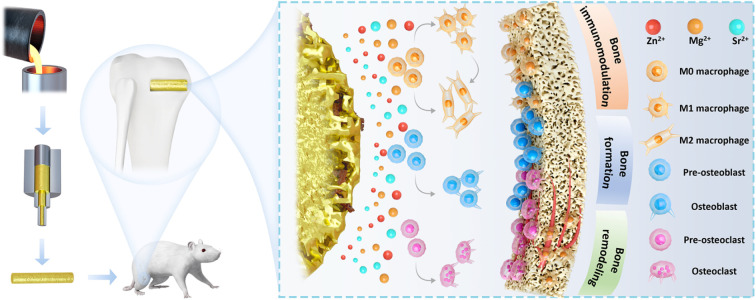

## 1. Introduction

Internal fixation materials, also known as osteosynthesis implants, are commonly used in the surgical treatment of bone fractures and deformities. These devices, including plates, screws, nails, and rods, are designed to provide mechanical support, maintain proper bone alignment, and facilitate the healing process. Currently, most internal fixation materials used in clinical practice are made of non-degradable metals, primarily stainless steel (316L) and titanium (Ti) alloys. Although these materials offer high mechanical strength and acceptable biocompatibility, they often remain in the body permanently or require a secondary surgery for removal after bone fracture healing, which increases patient's burden and raises the risk of infection.^1,2^ Furthermore, due to their lack of bioactivity, conventional internal fixation materials may have limited ability to actively promote bone healing, thereby increasing the risk of complications such as delayed union or nonunion.^3^

Considering these limitations, biodegradable metals (BMs) have emerged as promising alternatives for internal fixation.^4^ Unlike traditional internal fixation materials, BMs can be gradually absorbed by the host, allowing complete bone regeneration without the need for secondary removal surgery.^5^ To date, research on BMs for medical applications has primarily focused on magnesium (Mg), iron (Fe), and zinc (Zn) systems, including their alloys and composite derivatives.^6–8^ Although Mg-based alloys represent the most clinically developed biodegradable metals, their rapid corrosion rate in physiological environments can result in the premature loss of mechanical integrity, as well as the accumulation of hydrogen gas, which hinders their clinical application.^9,10^ Fe-based alloys exhibit an excessively slow corrosion rate, leaving non-absorbable residues that may hinder bone healing and cause potential long-term complications.^11^ Zn exhibits moderate corrosion behavior between that of Fe and Mg, with a standard electrode potential (−0.76 V) positioned between that of Fe (−0.44 V) and Mg (−2.37 V).^12,13^ Moreover, Zn degradation is characterized by the absence of gas evolution and the formation of biocompatible degradation products that can be effectively metabolized.^14,15^ In addition, Zn functions as an essential trace element that participates in critical cellular processes and as a bioactive agent that stimulates osteogenesis.^16–18^ These advantages highlight the clinical potential of Zn and have drawn growing interest from the academic community.

Nevertheless, pure Zn is not suitable for direct use as a standalone internal fixation material due to its inherent limitations. One key issue is its insufficient mechanical strength, which prevents Zn from offering adequate structural support for bone stabilization, particularly at weight-bearing sites.^19^ Moreover, Zn is prone to pitting corrosion when exposed to chloride ions in body fluids.^20^ This uncontrolled degradation may lead to rapid material loss and irregular structural damage, ultimately resulting in mechanical failure. The burst release of Zn^2+^ associated with pitting corrosion increases the risk of toxicity, potentially delaying bone healing or even inducing tissue necrosis. To address these issues, alloying Zn with other biocompatible elements, such as strontium (Sr) and Mg, has been proposed as an effective strategy to improve mechanical performance and corrosion behavior by microstructure refinement, intermetallic phases formation, and passivation regulation.^21,22^ More importantly, these alloying elements may induce additional biofunctionalities. For instance, Mg^2+^ enhances osteogenesis by activating the Wnt signaling pathway and stimulating the secretion of calcitonin gene-related polypeptide-α (CGRP) from peripheral neurons.^23,24^ Additionally, Sr^2+^ has been demonstrated to enhance bone formation by promoting the proliferation and differentiation of osteoblasts.^25–28^

In our previous research,^29^ we developed a Zn-0.8Mg-0.2Sr ternary alloy with superior mechanical properties (ultimate tensile strength = 324 MPa, tensile yield strength = 244 MPa, and microhardness = 87), compared to pure Zn, fulfilling the requirements for orthopedic fixation devices. However, an ideal bone implant material must possess not only sufficient mechanical strength but also excellent biocompatibility, controlled biodegradability, and satisfactory biofunctionality. Following our initial pilot studies,^30,31^ this work aimed to systematically evaluate the biological performance of Zn-0.8Mg-0.2Sr under an osteogenic microenvironment through both *in vitro* and *in vivo* experiments, and to further validate its feasibility as a biodegradable internal fixation material.

## 2. Materials and methods

### 2.1 Materials preparation

Zn-0.8Mg-0.2Sr (wt%) was prepared according to previous methods.^29^ Briefly, high-purity elemental Zn, Mg, and Sr were melted at 520 °C under ambient conditions with continuous stirring for 20 minutes, followed by casting, annealing at 350 °C for 24 hours, and water quenching. The resulting homogenized ingots were machined into billets (*Ø*30 × 35 mm) and hot-extruded at 200 °C with an extrusion ratio of 25 : 1. Specimens were then fabricated in two geometries: discs (*Ø*5.5 × 1 mm) for *in vitro* studies and cylinders (*Ø*2 × 4 mm) for *in vivo* implantation. Pure Zn samples (Zhong Ke Yan Nuo New Materials Co., Ltd., Beijing, China) with the same dimensions were processed as controls. All samples underwent sequential surface grinding, ultrasonic cleaning in acetone and ethanol for 15 minutes each, and UV sterilization for 2 hours before biological assessments, as described in our previous study.^32^

### 2.2 Microstructure analysis

The microstructure of Zn-0.8Mg-0.2Sr was characterized using scanning electron microscopy (SEM, Tescan Vega 3 LMU) coupled with energy-dispersive X-ray spectroscopy (EDS, Oxford Instruments Aztec) and electron backscatter diffraction system (EBSD, Oxford Instruments Aztec).

### 2.3 *In vitro* corrosion behavior assessment

To evaluate the corrosion behavior, 72 hours static immersion and electrochemical corrosion tests were conducted. The experimental design was mainly based on ISO 10993-16 and ASTM standards G3, G59, and G96,^33–36^ with appropriate modifications to accommodate the specific requirements and physiological relevance of this study. To ensure consistency between the *in vitro* corrosion tests and the subsequent biological experiments, the corrosion tests were performed under the same conditions as cell culture, using α-minimum essential medium (α-MEM, Gibco) supplemented with 10% fetal bovine serum (FBS, Gibco) and 1% penicillin/streptomycin (Hyclone, USA) at 37 °C in a standard atmosphere. Environmental humidity was maintained by placing an open water reservoir in the testing chamber. To stabilize pH, the α-MEM medium was supplemented with a TES buffer system (2-{[1,3-dihydroxy-2-(hydroxymethyl)propan-2-yl]amino}ethane-1-sulfonic acid, 5.9 g L^−1^), and the pH was adjusted with NaOH to restore the original value.

Static corrosion tests were conducted using two surface-to-volume (S/V) ratios—0.25 cm^2^ mL^−1^ and 1 cm^2^ mL^−1^—in sterile 12-well plates (ThermoFisher Scientific). The extracts were subsequently stabilized by the addition of HNO_3_ and analyzed using a flame atomic absorption spectrometer (AAS, GBC SavantAA). Surface morphology was examined by SEM equipped with EDS. Electrochemical corrosion tests were performed in sterile 25 mL tubes (Eppendorf) at an S/V ratio of 0.25 cm^2^ mL^−1^ using a three-electrode setup with a silver/silver chloride reference electrode (SSCE, internal electrolyte: 3.5 mol per L KCl) and a glassy carbon counter electrode. Electrochemical measurements included open-circuit potential (*E*_OC_) and polarization resistance (*R*_P_) monitoring every six hours (±10 mV per *E*_OC_, scan rate: 0.125 mV s^−1^). At the end of the exposure, potentiodynamic polarization curves were recorded in both directions: cathodic (0.05 V per *E*_OC_ to −2 V per SSCE) and anodic (−0.05 V per *E*_OC_ to −0.5 V per SSCE) at a scan rate of 1 mV s^−1^. All electrochemical corrosion tests were conducted using a Gamry Reference 600 system equipped with an ECM8 multiplexer, and data were analyzed using an Echem Analyst software.

### 2.4 Cytocompatibility analysis

#### 2.4.1 Cell culture

The mouse osteoblastic cell line MC3T3-E1 (Cell Bank of the Chinese Academy of Sciences) was used *in vitro*. Cells were cultured in α-MEM supplemented with 10% FBS and 1% penicillin/streptomycin at 37 °C in an incubator with 5% CO_2_ and 95% humidity. Cells were harvested at 80–90% confluence using 0.25% trypsin (Gibco, USA), followed by centrifugation at 1000 rpm for 5 minutes and resuspension for subsequent experiments.

#### 2.4.2 Preparation of material extracts

Material extracts were prepared by immersing Zn-0.8Mg-0.2Sr and pure Zn discs in α-MEM containing 10% FBS for 72 hours under conditions of 37 °C, 5% CO_2_, and 95% humidity according to ISO 10993-12:2021.^37^ All extracts were filter-sterilized using a 0.22 μm membrane filter (Millipore, Germany). Metal ion concentrations in the extracts were quantified using an inductively coupled plasma optical emission spectrometer (ICP-OES, PerkinElmer Avio 200, USA). Based on the ICP-OES results, the original extracts of pure Zn and Zn-0.8Mg-0.2Sr were normalized to an equal Zn^2+^ concentration using blank medium. The normalized extracts of pure Zn and Zn-0.8Mg-0.2Sr were prepared in serial dilutions (100%, 50%, 25%, 10%, and 5%) for cellular experiments. The cytocompatibility assessment was conducted according to ISO 10993-5:2009.^38^ The extracts of pure titanium (Ti) and copper (Cu) discs (Zhong Ke Yan Nuo New Materials Co., Ltd., Beijing, China) were included as negative and positive controls, respectively.

#### 2.4.3 Cell morphology observation

For morphological observation, MC3T3-E1 cells (1 × 10^5^ cells per well) were cultured in confocal dishes for 24 h, followed by treatment with the extract solutions (100%, 50%, and 25%) for an additional 24 hours. Cells were then fixed with 4% paraformaldehyde (PFA, Biosharp, China), permeabilized with 0.1% Triton X-100, and stained with FITC-phalloidin and DAPI (Solarbio, China) to visualize the cytoskeleton and nuclei. Fluorescence images were captured using a confocal laser scanning microscope (CLSM, Olympus, Japan).

#### 2.4.4 Live/dead staining assay

Cytotoxicity was assessed *via* a live/dead staining assay. MC3T3-E1 cells (1 × 10^5^ cells per well) were cultured in 24-well plates for 24 hours, followed by treatment with the extract dilutions (100%, 50%, and 25%) for an additional 24 hours. Cells were then stained with 200 μL of staining solution containing 2 μM Calcein-AM and 4.5 μM PI (Solarbio, China). Fluorescence images of stained cells were captured using a fluorescence microscope (Leica, Germany).

#### 2.4.5 Cell viability assessment

Cell viability was quantitatively assessed using a Cell Counting Kit-8 (CCK-8, Dojindo, Japan). Briefly, MC3T3-E1 cells (1 × 10^4^ cells per well) were cultured in 96-well plates for 24 hours, followed by treatment with 100 μL of extract dilutions (100%, 50%, and 25%) for an additional 24 hours. Subsequently, the medium was replaced with 100 μL of fresh medium and 10 μL of CCK-8 reagent. After a 2 hours incubation, the optical density (OD) value at 450 nm was measured using a microplate reader (SpectraMaxiD5, Molecular Devices, USA).

### 2.5 *In vitro* osteogenic differentiation evaluation

#### 2.5.1 Induction of osteogenic differentiation

MC3T3-E1 cells (1 × 10^5^ cells per well) were cultured in 6-well plates for 24 hours. The medium was then replaced with osteogenic induction medium, consisting of complete α-MEM supplemented with 10 mM β-glycerophosphate, 0.25 mM ascorbate, and 100 nM dexamethasone, along with material extract dilutions (5%, 10%, and 25%).

#### 2.5.2 Alkaline phosphatase (ALP) staining and quantitative assay

After 7 days of osteogenic induction, ALP expression was assessed using a BCIP/NBT Kit (Beyotime, China) and quantified *via* an ALP Assay Kit (Beyotime, China) according to the manufacturer's protocols. The stained samples were visualized using an optical microscope (Olympus, Japan), and the enzymatic activity was measured by a microplate reader at a wavelength of 405 nm.

#### 2.5.3 Osteopontin (OPN) immunofluorescence staining

After 14 days of osteogenic induction, OPN expression was evaluated *via* immunofluorescence. Briefly, cells were fixed with 4% PFA for 10 minutes, permeabilized with 0.1% Triton X-100 for 5 minutes, and blocked with 1% bovine serum albumin (BSA) for 30 minutes. Subsequently, the cells were incubated with an anti-OPN primary antibody (ab63856, Abcam), followed by sequential incubation with fluorescent secondary antibodies, phalloidin, and DAPI. Fluorescence images of the stained cells were captured using a CLSM, and fluorescence intensity was quantified utilizing ImageJ software (v1.54f, NIH, USA).

#### 2.5.4 Alizarin Red S (ARS) staining and semi-quantitative assay

After 14 days of osteogenic induction, ARS staining was conducted using an Osteogenesis Assay Kit (Beyotime, China) to evaluate mineralization. The calcium deposits stained with ARS were observed under an optical microscope. For semi-quantitative analysis, the stained calcium nodules were eluted with a cetylpyridinium chloride solution (Solarbio, China) for 20 minutes. The resulting solution was collected and measured by a microplate reader at a wavelength of 560 nm.

### 2.6 Animal study

The animal study was conducted using a rat tibial defect model,^39,40^ approved by the Ethics Committee of West China Hospital of Stomatology (WCHSIRB-D-2020-006), and carried out at the Animal Experiment Center of West China Hospital following the NIH guidelines for animal welfare. Thirty male Sprague-Dawley (SD) rats aged 10 weeks were obtained from the Laboratory Animal Centre of Sichuan University and randomly assigned to three groups: the sham group (surgery without implantation, *n* = 4), the Zn group (*n* = 13), and the Zn-0.8Mg-0.2Sr group (*n* = 13).

#### 2.6.1 Surgery

Rats were anesthetized with isoflurane, administered and maintained *via* continuous inhalation through a mask during surgery. After hair removal, disinfection, and local anesthesia of the surgical area, a 2 cm longitudinal incision was made on the inner knee to expose the anteromedial tibial metaphysis. A cylindrical defect (*Ø*2 × 4 mm) was created by drilling under saline cooling. Each cylindrical implant (*Ø*2 × 4 mm) was inserted and the wounds were closed with absorbable sutures. Postoperative care included penicillin prophylaxis and wound monitoring. Four weeks after surgery, all animals were euthanized by carbon dioxide asphyxiation, and tibial segments containing the implants were harvested for subsequent analyses.

#### 2.6.2 Systemic toxicity assessment

After euthanasia, blood samples were collected to measure the concentrations of Zn^2+^, Mg^2+^, and Sr^2+^ using inductively coupled plasma mass spectrometry (ICP-MS, Thermo Scientific iCAP™ RQ, USA). Moreover, major organs (heart, liver, spleen, lungs, and kidneys) were harvested, fixed in 4% PFA, and then processed for hematoxylin and eosin (H&E) staining and histological evaluation.

#### 2.6.3 *In vivo* corrosion and degradation observation

To document the morphology and adherent tissue on the implant surfaces, implants extracted from the tibial tissue were immediately examined using a stereomicroscope. Representative images of the lateral and basal surfaces of the cylindrical implants were captured at 20× and 35× magnifications, respectively. Furthermore, the tibial specimens containing the implants were embedded in acrylic resin (Technovit 7200, Kulzer, Germany) and sectioned at the midpoint. The exposed surface was then ground, polished, and sputter-coated with gold. The elemental distribution at the bone–implant interface was analyzed using an electron probe microanalyzer (EPMA, JEOL JAX-8230, Japan) equipped with a wavelength-dispersive X-ray spectrometer (WDS) under an accelerating voltage of 15 kV. Elemental mapping for C, O, P, Ca, Zn, Mg, Sr, and Cl was performed at both overall (40×) and localized (400×) scales.

#### 2.6.4 Micro-CT analysis

The tibial specimens containing metallic implants (*n* = 3) were scanned using a Micro-CT system (μCT 50, Scanco Medical AG, Switzerland) at a resolution of 7 μm with 500 projections over 180° (voltage = 90 kV, current = 200 μA). Bone and implant components were segmented using grayscale thresholds of 80 and 180, respectively. Parameters of bone regeneration, including bone volume fraction (BV/TV), trabecular number (Tb.N), trabecular thickness (Tb.Th), and trabecular separation (Tb.Sp) were analyzed within a 1 mm peri-implant region of interest (ROI) *via* the manufacturer's software.

#### 2.6.5 Histological evaluations

To further evaluate the bone-implant interface, tibial specimens containing implants (*n* = 3) were prepared as undecalcified hard tissue sections for histological staining. Briefly, tissue samples fixed in 4% PFA were subjected to graded dehydration and embedded in acrylic resin. The resin blocks were sectioned into 200 μm slices using a hard tissue slicer (EXAKT300CP, Germany), followed by grinding and polishing to a final thickness of 20 μm using a polishing grinder (EXAKT400S, Germany). After H&E and Goldner's trichrome staining, the sections were observed by a light microscope. The contours of implants were analyzed using ImageJ software.

In addition to hard tissue sections, decalcified paraffin-embedded sections were prepared to better assess peri-implant tissues and enable subsequent immunohistochemical analysis. For decalcified sections, the metallic implants were removed prior to fixation in 4% PFA. Tibias without implants (*n* = 3) were then decalcified in 10% EDTA solution (Solarbio, China) for 45 days. After decalcification, samples were embedded in paraffin and then sectioned into 3 μm slices using a microtome (Leica RM2016, Germany), followed by H&E and Masson's trichrome staining. ImageJ software was used to quantify the area of new bone and mature bone (stained purple in Masson's trichrome). Additionally, tartrate-resistant acid phosphatase (TRAP) staining was conducted to evaluate osteoclastic activity around the implants.

#### 2.6.6 Immunohistochemistry assessment

To assess peri-implant immune responses, sections underwent antigen retrieval for 20 minutes and blocking in 5% BSA for 30 minutes, followed by overnight incubation at 4 °C with primary antibodies against CD68 (a pan-macrophage marker; Abcam, ab283654), iNOS (an M1 macrophage marker; Abcam, ab283655), and CD163 (an M2 macrophage marker; Abcam, ab182422). After washing with PBS, HRP-conjugated secondary antibodies and DAB chromogen were applied for visualization under a light microscope.

#### 2.6.7 Quantitative real-time polymerase chain reaction (qRT-PCR)

qRT-PCR was performed to evaluate gene expression related to macrophage polarization and osteogenic differentiation. After implant removal, adjacent tibial tissues (*n* = 4) were pulverized in liquid nitrogen using a mortar. Total RNA was extracted using an MasterPure™ Complete RNA Purification Kit (Epicentre, USA) and reverse-transcribed into cDNA using a PrimeScript™ RT reagent (TaKaRa, Japan). Glyceraldehyde-3-phosphate dehydrogenase (Gapdh) was used as reference gene and the sham operation group as blank control, qRT-PCR was conducted on a LightCycler®96 (Roche, USA) with TB Green® Premix Ex Taq™ II (TaKaRa, Japan). After amplification, the quantification cycle (Cq) value for was obtained. The relative expression levels of *Nos2* (M1), *Arg1* (M2), and osteogenic-related genes (Runx2, Alpl, and *Bmp2*) were calculated using the 2^−ΔΔCt^ method. Primer sequences are listed in Table S1.

### 2.7 Statistical analysis

Statistical analysis was performed using GraphPad Prism 9.5. After verifying data normality and homogeneity of variance, appropriate statistical tests were applied: independent *t*-tests for two-group comparisons and one-way ANOVA with Tukey's *post hoc* analysis for multiple-group comparisons. Statistical significance was indicated as *p < 0.05, **p < 0.01, ***p < 0.001, and ****p < 0.0001. All biological data were obtained from at least three independent replicates.

## 3. Results and discussion

### 3.1 Microstructure of Zn-0.8Mg-0.2Sr alloy

**Fig. 1:**
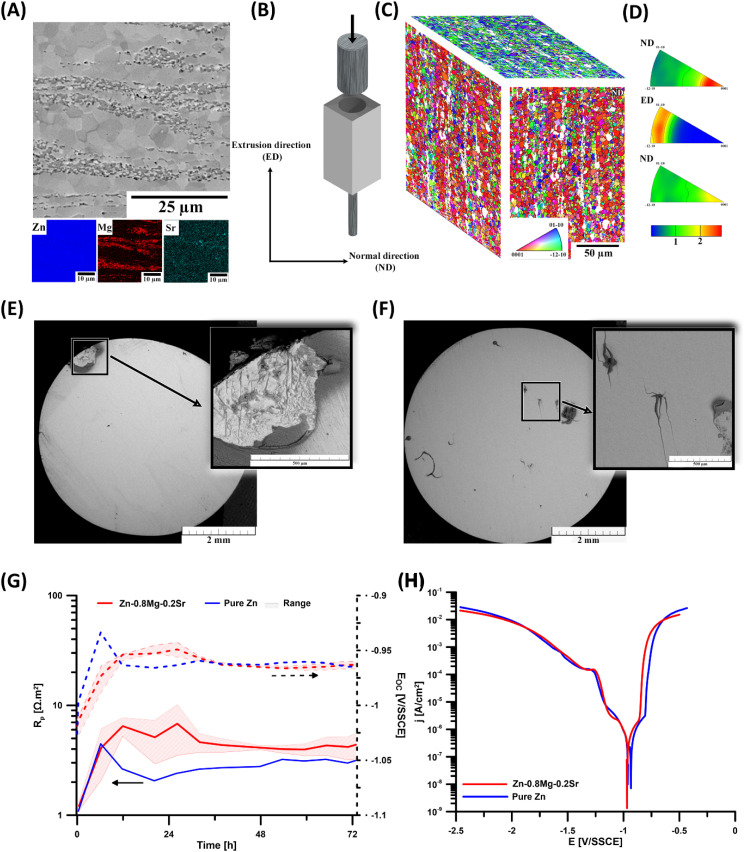
Microstructure and *in vitro* corrosion behavior of Zn-0.8Mg-0.2Sr. (A) SEM micrograph and EDS elemental maps of Zn-0.8Mg-0.2Sr; (B) Scheme of the extrusion process and designation of the sample directions; (C) inverse pole figure maps of Zn-0.8Mg-0.2Sr (EBSD data were acquired from the longitudinal section – ND sample direction and evaluated for all three main sample directions – ND1, ND2, and ED); (D) inverse pole figure of Zn-0.8Mg-0.2Sr; (E) and (F) macrographs of corroded Zn and Zn-0.8Mg-0.2Sr, respectively; (G) dependence of polarization resistance and open circuit potential over time; (H) potentiodynamic curves of the investigated materials.

As shown in Fig. 1A, the Zn-0.8Mg-0.2Sr alloy consists of a Zn matrix with equiaxed recrystallized grains (approximately 2.5 μm), characteristic of hot-worked materials.^41^ Besides the Zn matrix, intermetallic particles are aligned in rows parallel to the extrusion direction (Fig. 1B). These intermetallic particles are predominantly composed of Mg_2_Zn_11_ and SrZn_13_,^29,42^ resulting from the Mg and Sr content exceeding their solubility limits in Zn. At the nominal composition, both intermetallic phases are thermodynamically stable and expected based on the selected binary phase diagrams.^43,44^ The inverse pole figure maps (Fig. 1C) and inverse pole figures (Fig. 1D) show a characteristic texture with basal planes aligned nearly parallel to the extrusion direction, a typical texture of extruded Zn-based materials resulting from combined basal and second-order pyramidal <*c* + *a*> slip during extrusion.^45^

As previously reported, the presence of intermetallic particles and the fine-grained microstructure significantly enhances the alloy's strength by hindering dislocation slip and suppressing twinning.^41,46,47^ This microstructural refinement, along with the characteristic texture, is likely to contribute to the mechanical performance of Zn-0.8Mg-0.2Sr alloy, supporting its suitability as a bone implant material.

### 3.2 Zn-0.8Mg-0.2Sr exhibited more uniform corrosion behavior *in vitro*

Static corrosion tests were performed for 72 hours at two different surface-to-volume (S/V) ratios. The first ratio (0.25 cm^2^ mL^−1^) was the same as that used in the electrochemical corrosion tests, while the second ratio (1 cm^2^ mL^−1^) was chosen to simulate the limited ion exchange in the bone implant region. At the lower S/V ratio, the Zn^2+^ concentration detected in the Zn-0.8Mg-0.2Sr group was 28.75 ± 0.57 μg mL^−1^, accounting for approximately 28% of the amount detected in the pure Zn group. Based on these values, the calculated corrosion rate under uniform dissolution conditions was 20 μm·a^−1^. At the higher S/V ratio, the Zn^2+^ concentration significantly increased to 36.3 μg mL^−1^ (*p* = 0.015). Visual assessment (Fig. S1) revealed that Zn-0.8Mg-0.2Sr appeared more greyish than pure Zn, while still maintaining a metallic luster. Small white fibrous deposits were observed on all materials and appeared with reduced BSE intensity under SEM (Fig. 1E, F, S2, and S4). Compared with pure Zn, these deposits appeared more frequently and with smaller particle sizes on Zn-0.8Mg-0.2Sr. Extensive areas of visible corrosion were occasionally observed on the alloy. EDS analysis (Tables S2 and S4) indicated that the fibrous precipitates were mainly composed of C and O, along with Zn, P, and Ca. For the alloy, Mg and trace amounts of S, Cl, and occasionally K were also detected. Based on morphology and elemental composition, these deposits were presumed to be proteinaceous and secondarily mineralized by inorganic ions from the medium. On the “bare” metal surfaces, Zn-0.8Mg-0.2Sr exhibited higher P/Zn and O/Zn ratios compared to pure Zn. Analysis of the corrosion products after mechanical removal of the fibrous deposits revealed an increased presence of P, along with notable Cl and S. The presence of Zn, P, O, and Cl corresponds to the insoluble corrosion products Zn_3_(PO_4_)_2_·4H_2_O and Zn_5_(OH)_8_Cl_2_ from thermodynamic simulations (Fig. S8). The presence of S, probably derived from cysteine or cystine, also suggests the involvement of an organic component.

To evaluate corrosion kinetics, an *E*_OC_-*R*_P_ trend was investigated (Fig. 1G). In the Zn-0.8Mg-0.2Sr group, a marked increase in *R*_P_ during the first 12 hours indicated the formation of a corrosion-inhibiting barrier. This was followed by a stabilization phase, reaching a steady-state *R*_P_ of approximately 4 Ω m^2^ over the next 20 hours. Pure Zn exhibited a similar initial trend within the first 6 hours. However, its corrosion rate subsequently accelerated, peaking at 12 hours, and then gradually slowed, stabilizing at ∼3 Ω m^2^. These *R*_P_ trends were consistent with the evolution of *E*_OC_. Pure Zn showed a more rapid initial increase and reached a higher peak potential (−0.93 V per SSCE, *i.e.* −0.72 mV per SHE). After 12 hours, both materials stabilized at −0.96 to −0.97 V per SSCE (*i.e.*, −0.75 to −0.76 V per SHE). Potentiodynamic polarization curves (Fig. 1H) further supported the formation of passivation layers. For the alloy, a Tafel slope of 186 mV dec^−1^ was determined in the anodic linear region above the corrosion potential, spanning from −0.91 V per SSCE to −0.88 V per SSCE. Beyond this range, the current increased by more than 3 orders of magnitude, causing significant pitting of the material. On the cathodic branch, a linear region between −0.98 V per SSCE and −1.06 V per SSCE was observed, with a Tafel slope ranging from 184–203 mV dec^−1^, indicating the presence of reducible species beyond oxygen depolarization. Furthermore, the cathodic slope for Zn-0.8Mg-0.2Sr decreased further toward the limiting current plateau. At −1.11 V per SSCE for Zn-0.8Mg-0.2Sr and −1.16 V per SSCE for pure Zn, a new wave appeared, with half-wave potentials of −1.18 V per SSCE and −1.20 V per SSCE for pure Zn and Zn-0.8Mg-0.2Sr, respectively. The limiting current plateau reappeared at −1.26 V per SSCE, and hydrogen evolution began at −1.35 V per SSCE. The Stern–Geary coefficient (0.04) derived from the obtained Tafel slopes was used to estimate the corrosion rate. Assuming uniform dissolution from the resulting polarization resistance values, the calculated corrosion rate for Zn-0.8Mg-0.2Sr was 14 ± 4 μm·a^−1^, approximately 70% of pure Zn.

Combined with impedance analysis (Fig. S5–S7), these findings suggest that the corrosion of Zn-0.8Mg-0.2Sr likely initiates at intermetallic phases, promoting a more uniform localized attack. Furthermore, the formation of organic-rich deposits may regulate local metal ion concentrations, thereby leading to precipitation of insoluble products (*e.g.*, Zn or Mg phosphates) and ultimately limiting the corrosion rate at these sites.

### 3.3 The cytotoxicity of Zn-0.8Mg-0.2Sr exhibited a dose-dependent manner

**Fig. 2:**
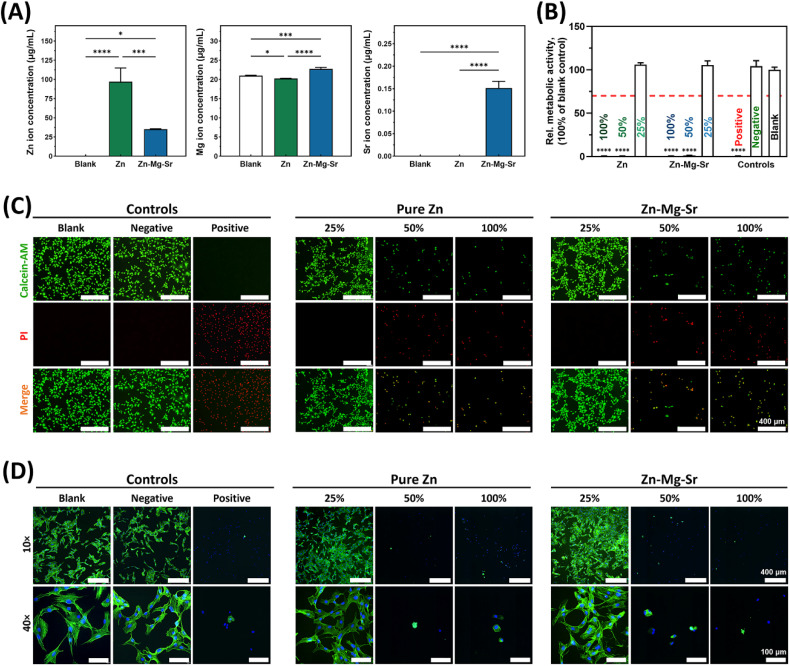
The cytocompatibility assessment of Zn-0.8Mg-0.2Sr and pure Zn. (A) Ion concentrations of Zn^2+^, Mg^2+^, and Sr^2+^ in each group after incubation in α-minimum essential medium containing 10% fetal bovine serum under cell culture conditions for 72 h (*n* = 3); (B) relative metabolic activities, (C) live/dead staining, and (D) cytoskeletal staining of MC3T3-E1 pre-osteoblasts cultured in various dilutions (100%, 50%, and 25%) of pure Zn and Zn-0.8Mg-0.2Sr extracts. Ti and Cu were used as negative and positive controls, respectively. The red dashed line in (B), set at 70% of the blank control, indicates the threshold for distinguishing cytotoxic from non-cytotoxic effects, according to ISO standard. Asterisks indicate statistically significant differences (*p < 0.05, ***p < 0.001, and ****p < 0.0001).

The concentrations of Zn^2+^, Mg^2+^ and Sr^2+^ in the blank medium, pure Zn extracts, and Zn-0.8Mg-0.2Sr extracts are shown in Fig. 2A and Tables S5–S7. Zn^2+^ levels in the original extracts of pure Zn (97.03 ± 17.93 μg mL^−1^) and Zn-0.8Mg-0.2Sr (35.20 ± 0.60 μg mL^−1^) were significantly higher than those in the blank medium (0.24 ± 0.01 μg mL^−1^). Notably, Zn^2+^ amount released from Zn-0.8Mg-0.2Sr was significantly lower than that from pure Zn. Mg^2+^ levels in the original extracts of Zn-0.8Mg-0.2Sr (22.73 ± 0.42 μg mL^−1^) were significantly higher than those of pure Zn (20.23 ± 0.06 μg mL^−1^) and blank medium (20.97 ± 0.15 μg mL^−1^). Interestingly, the Mg^2+^ level in the original extracts of pure Zn showed a slight decrease compared to that in the blank medium, possibly due to partial adsorption of Mg^2+^ from the culture medium onto the surface of pure Zn during immersion. This hypothesis can be supported by the SEM-EDS analysis, which detected trace amounts of Mg on the surface of pure Zn after immersion (Table S4). Sr^2+^ levels in the blank medium and pure Zn extracts were undetectable as they were below the minimum detection limit of ICP-OES, while Sr^2+^ concentration in the original extracts of Zn-0.8Mg-0.2Sr could be detected with a value of 0.15 ± 0.02 μg mL^−1^. To enable a valid comparison of biological effects of the individual alloying elements, Zn^2+^ concentrations in the pure Zn and Zn-0.8Mg-0.2Sr extracts were normalized to the same value (35.2 μg mL^−1^). Then, the normalized extracts were serially diluted (100%, 50%, 25%, 10%, and 5%) for subsequent cellular assays. The concentrations of Zn^2+^, Mg^2+^, and Sr^2+^ in each dilution are listed in Tables S6 and S7.

CCK-8 assay results (Fig. 2B) revealed that 100% and 50% extracts of pure Zn and Zn-0.8Mg-0.2Sr significantly reduced the viability of MC3T3-E1 cells, corresponding to Grade 4 cytotoxicity, as defined by ISO 10993-5:2009.^38^ Consistently, live/dead staining (Fig. 2C) showed that majority of MC3T3-E1 cells exposed to 100% and 50% extracts underwent cell death (red fluorescence). In contrast, cells cultured in 25% extracts remained predominantly viable (green fluorescence), with no significant difference compared to the blank and negative controls. Cytoskeletal staining results (Fig. 2D) provided further confirmation: MC3T3-E1 cells treated with 100% and 50% extracts exhibited a rounded shape without filopodia, resembling cells in the positive control. Conversely, cells exposed to 25% extracts showed polygonal morphology and well-organized filamentous F-actin structures, comparable to those observed in the blank and negative controls.

The observed cytotoxicity trend resulted from Zn^2+^ concentration-dependent cellular response: at low concentrations, Zn^2+^ promotes cell viability, proliferation, and migration; however, higher levels of Zn^2+^ induce mitochondrial fission, disturb mitochondrial dynamics, and disrupt intracellular redox homeostasis, ultimately causing oxidative stress-induced cell death.^48–50^ In this study, Zn^2+^ levels in 100% and 50% extracts exceeded the cellular tolerance threshold, while 25% extracts maintained non-toxic levels. This concentration-dependent pattern is consistent with previous findings. For example, Tong *et al.* reported maximal MC3T3-E1 viability at 25% Zn-5La alloy extracts.^51^ Similarly, Ma *et al.* found that endothelial cell proliferation was inhibited at Zn^2+^ concentrations above 5.2 μg mL^−1^, and cell viability declined significantly above 6.5 μg mL^−1^.^52^ In this study, Zn^2+^ concentrations in 25% extracts (8.98 μg mL^−1^) exceeded 6.5 μg mL^−1^ but induced no cytotoxic effects on MC3T3-E1 cells, likely due to cell type-specific tolerance thresholds to Zn^2+^.^53^

### 3.4 The Zn-0.8Mg-0.2Sr extracts promoted osteogenic differentiation at non-toxic levels *in vitro*

**Fig. 3:**
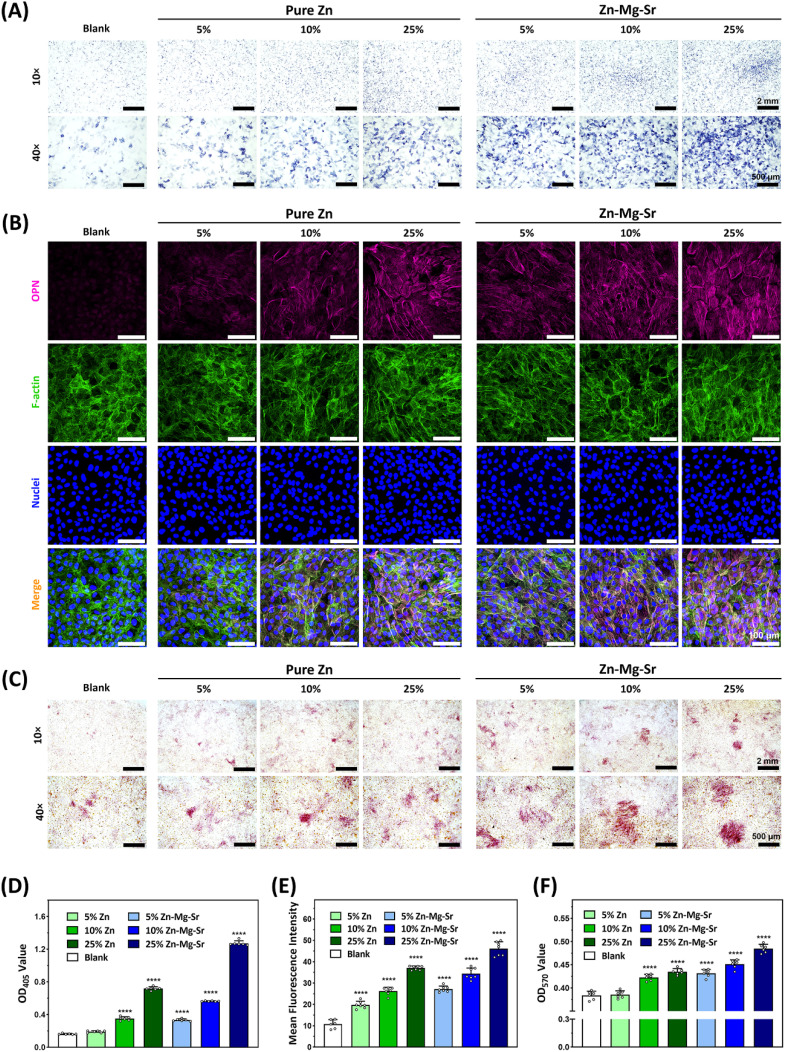
Effects of different extracts (5%, 10%, and 25%) of Zn and Zn-0.8Mg-0.2Sr on the osteogenic differentiation of MC3T3-E1 preosteoblasts in the early, intermediate and late stages of osteogenesis. Early stage: (A) alkaline phosphatase (ALP) staining and (D) quantitative analysis of ALP activity; intermediate stage: (B) immunofluorescence staining and (E) fluorescence intensity analysis of osteopontin (OPN); late stage: (C) alizarin red S (ARS) staining and (F) quantitative analysis of mineralized nodules stained by ARS. Asterisks indicate statistically significant differences compared to the blank control (****p < 0.0001).

Within the non-cytotoxic concentration range, the effects of different dilutions (5%, 10%, and 25%) of pure Zn and Zn-0.8Mg-0.2Sr on osteogenic differentiation of MC3T3-E1 cells were evaluated at early, intermediate, and late stages of osteogenesis. At day 7, ALP expression—a key early osteogenic marker—was assessed by staining and enzymatic activity measurements. Fig. 3A and D show that ALP expression in both groups increased with extract concentration; at equivalent concentrations, Zn-0.8Mg-0.2Sr consistently exhibited stronger ALP expression than pure Zn. At day 14, immunofluorescence staining of OPN, an intermediate-stage marker, revealed higher fluorescence intensity in the Zn-0.8Mg-0.2Sr group (Fig. 3B and E). At day 21, ARS staining demonstrated more mineralized nodule formation in the Zn-0.8Mg-0.2Sr group (Fig. 3C and F).

The results indicated that, within non-toxic Zn^2+^ concentrations (≤8.98 μg mL^−1^), osteogenic differentiation was positively correlated with Zn^2+^ dose. More importantly, at equivalent Zn^2+^ concentrations, Zn-0.8Mg-0.2Sr consistently exhibited stronger osteoinductive effects at all stages than pure Zn. This enhanced performance is likely attributable to the elevated levels of Mg^2+^ and Sr^2+^ in Zn-0.8Mg-0.2Sr extracts, which are known to synergistically promote osteogenesis beyond the effect of Zn^2+^ alone. Qin *et al.* reported that Zn-1Mg scaffolds exhibited superior osteogenic property compared to pure Zn.^54^ Additionally, Jia *et al.* demonstrated that Zn-Sr alloys enhanced osteogenesis by stimulating the proliferation and differentiation of MC3T3-E1, as well as up-regulating osteogenic genes and proteins such as ALP, COL I, OCN, and Runx-2.^19^

### 3.5 The Zn-0.8Mg-0.2Sr implants exhibited favorable biocompatibility *in vivo*

**Fig. 4:**
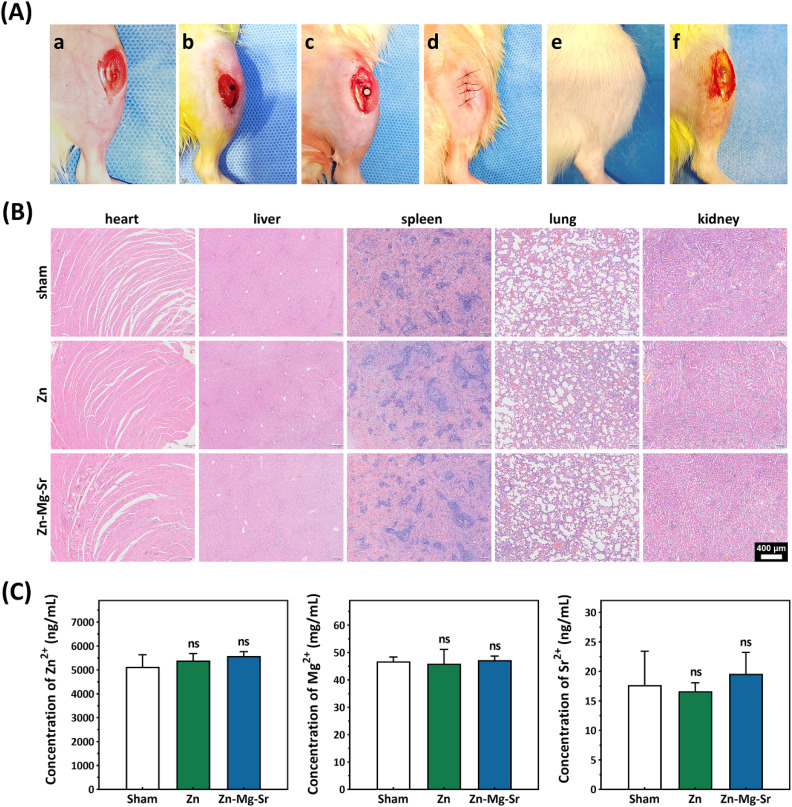
Systemic toxicity evaluation in rats following implantation of Zn and Zn-0.8Mg-0.2Sr rods into the tibial metaphysis for 28 days. (A) The surgical procedure of material implantation (a–d), the condition of wound healing (e), and re-exposure of the surgical site 28 days after surgery (f); (B) hematoxylin and Eosin (H&E) staining of rat heart, liver, spleen, lung, and kidney tissues from the sham, pure Zn, and Zn-0.8Mg 0.2Sr groups; (C) the ion concentrations of Zn^2+^, Mg^2+^, and Sr^2+^ in the blood of rats 28 days after material implantation (*n* = 4).

To investigate potential systemic toxicity, animal studies were conducted by implanting pure Zn and Zn-0.8Mg-0.2Sr rods into the tibial metaphysis of rats for 4 weeks (Fig. 4A(a–d)). After surgery, the surgical site healed well, with no signs of infection, hemorrhage, or tissue necrosis (Fig. 4A(e)). After 28 day implantation, the implants were surrounded by healthy bone tissue and covered by a dense periosteum (Fig. 4A(f)). Histopathological analysis of the major organs, including the heart, liver, spleen, lung, and kidney, exhibited neither morphological abnormalities nor inflammatory infiltration (Fig. 4B). In addition, no significant differences were observed in the concentrations of Zn^2+^, Mg^2+^, and Sr^2+^ in the blood between the experimental groups and the sham group 4 weeks post-implantation (Fig. 4C). These results indicate that both pure Zn and Zn-0.8Mg-0.2Sr alloy exhibit favorable *in vivo* biocompatibility, despite the apparent cytotoxicity of their high-concentrations extracts on MC3T3-E1 cells (Fig. 2). The discrepancy between *in vivo* and *in vitro* biocompatibility results could be attributed to the following factors: (1) the cytotoxicity evaluation in this research followed ISO standards 10993-5 and 10993-12, which were initially designed for non-absorbable materials; (2) the standardized *in vitro* assays failed to mimic the physiological metabolism and elimination processes occurring *in vivo*, which allow degradation products and released metal ions to be rapidly absorbed into the systemic circulation and subsequently either reutilized or excreted *via* feces and urine.^29,55^ These findings provide preliminary support for the potential clinical applicability of the Zn-0.8Mg-0.2Sr alloy, although further evaluation through large animal studies and long-term comprehensive assessments remains necessary.

### 3.6 The Zn-0.8Mg-0.2Sr implants exhibited controlled degradation *in vivo*

**Fig. 5:**
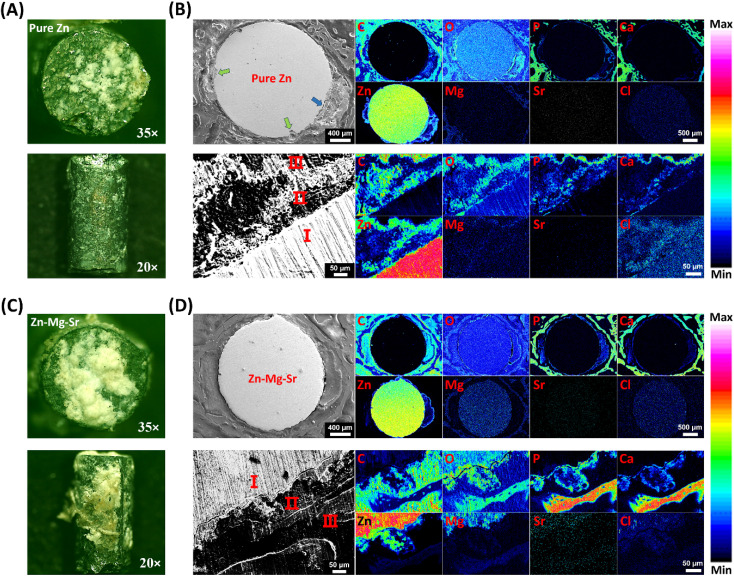
*In vivo* corrosion and degradation analysis of Zn and Zn-0.8Mg-0.2Sr implants after 28 days of implantation in tibial metaphysis of rats. (A) and (C) show representative stereomicroscopic images of extracted pure Zn and Zn-0.8Mg-0.2Sr, respectively; (B) and (D) display the cross-sectional morphology and elemental mappings of Zn and Zn-0.8Mg-0.2Sr implants, respectively. Different colors indicate the abundance of the investigated elements. Region I: metal matrix; region II: degradation products and fibrous connective tissue; region III: newly formed bone. The green and blue arrows in (B) indicate pitting corrosion and structural collapse, respectively.

The *in vitro* tests indicated that Zn-0.8Mg-0.2Sr exhibited milder corrosion behavior, whereas pure Zn exhibited more localized corrosion and potential pitting, which may result in structural damage and further compromise its inherently low mechanical strength. This finding was further confirmed by *in vivo* experiments. Fig. 5A and C show stereomicroscopic images of pure Zn and Zn-0.8Mg-0.2Sr implants extracted from tibia, respectively. After 4 weeks of implantation, the Zn-0.8Mg-0.2Sr surface displayed relatively uniform degradation products, tightly integrated with surrounding connective tissue, whereas that of pure Zn was mostly exposed metal. This difference might be attributed to the formation of a denser and more uniform degradation layer on the surface of Zn-0.8Mg-0.2Sr, which facilitated tissue anchorage, thereby preventing detachment during implant removal. To gain a deeper understanding of the degradation products around the implants, cross-sections of the bone–implant interface were examined by EPMA-WDS. As shown in the global images (Fig. 5B and D), the edges of the pure Zn implant partially maintained their original curvature, but pitting corrosion (green arrow) and structural collapse (blue arrow) were observed in certain areas, with corrosion depths ranging from approximately 40 to 88 μm. In contrast, the Zn-0.8Mg-0.2Sr implant displayed a more uniform corrosion pattern, with consistently jagged edges and no evidence of pitting corrosion or structural collapse. Furthermore, elemental analysis indicated that a significant amount of Zn was released at the sites of pitting corrosion and structural collapse, causing the detachment of surrounding newly formed bone from the pure Zn implant. In contrast, the concentration and area of Zn around the Zn-0.8Mg-0.2Sr implant were lower, and the newly formed bone remained at a relatively consistent distance from the implant surface.

For clarity, the bone-implant interface has been divided into three distinct regions: the metal matrix (region I), the degradation products and fibrous connective tissue (region II), and the newly formed bone rich in Ca and P (region III). In region I, in addition to the original metallic elements, trace amounts of O and Cl were detected. Region II of both implants primarily contained C, O, Zn. Notably, Mg and Sr were exclusively present around the Zn-0.8Mg-0.2Sr implant, and higher concentrations of P and Ca were observed in the degradation layer around the Zn-0.8Mg-0.2Sr implant. In the outermost region III, the newly formed bone adjacent to the Zn-0.8Mg-0.2Sr implant also exhibited greater Ca and P deposition. Presumably, the degradation products predominantly consist of zinc oxide, zinc hydroxide, zinc phosphate, zinc carbonate, and minor amount of chloride. This deduction could be supported by previous studies. Hybasek *et al.* discovered that degradation products of Zn-0.8Mg-0.2Sr in physiological saline comprised a mixture of oxides, hydroxides, and chlorides, whereas in α-MEM they predominantly comprised Zn_3_(PO_4_)_2_.^56^ Pinc *et al.* identified hydrozincite (Zn_5_(CO_3_)_2_(OH)_6_) and simonkolleite (Zn_5_(OH)_8_Cl_2_ H_2_O), along with minor amounts of Zn_*x*_ (PO_4_)_*y*_ as corrosion products of Zn-0.8Mg-0.2Sr in simulated body fluid (SBF).^42^ Importantly, compared to pure Zn, the increased phosphorus content in the degradation layer of Zn-0.8Mg-0.2Sr indicated enhanced formation of compounds such as zinc phosphate and magnesium phosphate during *in vivo* degradation. Previous studies have shown that these phosphates are crucial for improving the biocompatibility of zinc-based implants and facilitating tissue integration at the implant–tissue interface.^57,58^

### 3.7 The Zn-0.8Mg-0.2Sr implants showed enhanced *in vivo* bioactivities in promoting osteogenesis and modulating immune response

**Fig. 6:**
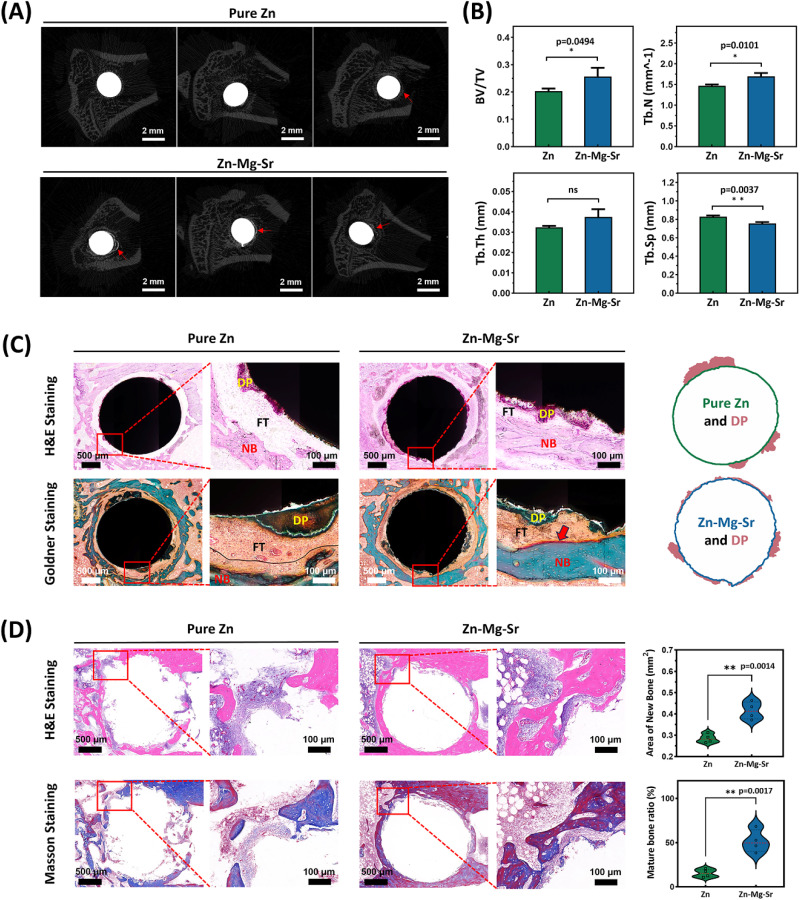
Analysis of bone-implant interface of pure Zn and Zn-0.8Mg-0.2Sr. (A) Micro-CT images. Red arrows indicate newly formed bone around the implants; (B) assessment of bone histomorphometric parameters (*n* = 3), including bone volume fraction (BV/TV), trabecular thickness (Tb.Th), trabecular number (Tb.N), and trabecular separation (Tb.Sp); (C) hematoxylin and Eosin (H&E) and Goldner's trichrome staining of hard tissue sections of undecalcified tibiae containing implants. Outlines and degradation products of pure Zn and Zn-0.8Mg-0.2Sr implants were extracted for comparison. DP, FT, and NB represent degradation products, fibrous tissue, and new bone, respectively; (D) H&E and Masson's trichrome staining of paraffin sections of decalcified tibiae without implants. Area of new bone and mature bone ratio were calculated for comparison (*n* = 4). Asterisks indicate statistically significant differences (*p < 0.05 and **p < 0.01).

Micro-CT scans were performed on rat tibiae after 28 day implantation. Fig. 6A displays representative cross-sectional images of the bone-implant interface. Compared to pure Zn, more pronounced hyperdense regions (red arrow) were observed around the Zn-0.8Mg-0.2Sr implant, indicating increased osteogenic activity and enhanced mineralization of newly formed bone. Quantitative analysis (Fig. 6B) demonstrated a higher bone volume fraction (BV/TV) and trabecular number (Tb.N), as well as lower trabecular separation (Tb.Sp) around the Zn-0.8Mg-0.2Sr implant. The enhanced osteogenic performance was further evidenced by H&E and Goldner's trichrome staining images of hard tissue sections (Fig. 6C). Compared to pure Zn, the surface of Zn-0.8Mg-0.2Sr implant was covered with a denser and more uniform degradation layer. Surrounding this layer, fibrous tissue (FT) was firmly attached to the metal surface in the absence of gas, and the FT layer around Zn-0.8Mg-0.2Sr displayed a denser cellular arrangement. Furthermore, a ring of new bone (NB) was observed around the FT layer. Interestingly, red-stained osteoid (red arrow) was only found at the FT-NB interface of Zn-0.8Mg-0.2Sr implant. H&E and Masson's trichrome staining of decalcified tibia tissues without implants (Fig. 6D) further confirmed that the new bone around Zn-0.8Mg-0.2Sr was dense, continuous, and formed a complete ring structure. In contrast, the bone surrounding pure Zn appeared discontinuous, with some regions lacking new bone formation. This observation was further supported by quantitative analysis, which showed that the new bone area around Zn-0.8Mg-0.2Sr was approximately 1.5 times greater than that around pure Zn. Moreover, Masson's trichrome staining revealed that the new bone around Zn-0.8Mg-0.2Sr was predominantly dark purple, whereas that surrounding pure Zn was mostly light blue, indicating a significantly higher degree of mineralization and maturation in the new bone around Zn-0.8Mg-0.2Sr. The superior osteogenic capability of Zn-0.8Mg-0.2Sr was attributed to its more uniform degradation behavior, which enabled the controlled release of Zn^2+^, preventing the cytotoxic effects associated with excessive local concentrations of Zn^2+^.^51,53^ Meanwhile, the synergistic effects of Mg^2+^ and Sr^2+^ may further enhance osteogenesis by promoting osteoblast differentiation and matrix mineralization.^19,54^ Beyond ionic effects, the degradation products on the implant surface may also contribute to new bone formation. Specifically, the P-rich degradation layer on Zn-0.8Mg-0.2Sr mentioned above might enhance bone formation through its excellent biocompatibility and bioactivity facilitating cell adhesion and tissue integration.^57–59^

**Fig. 7:**
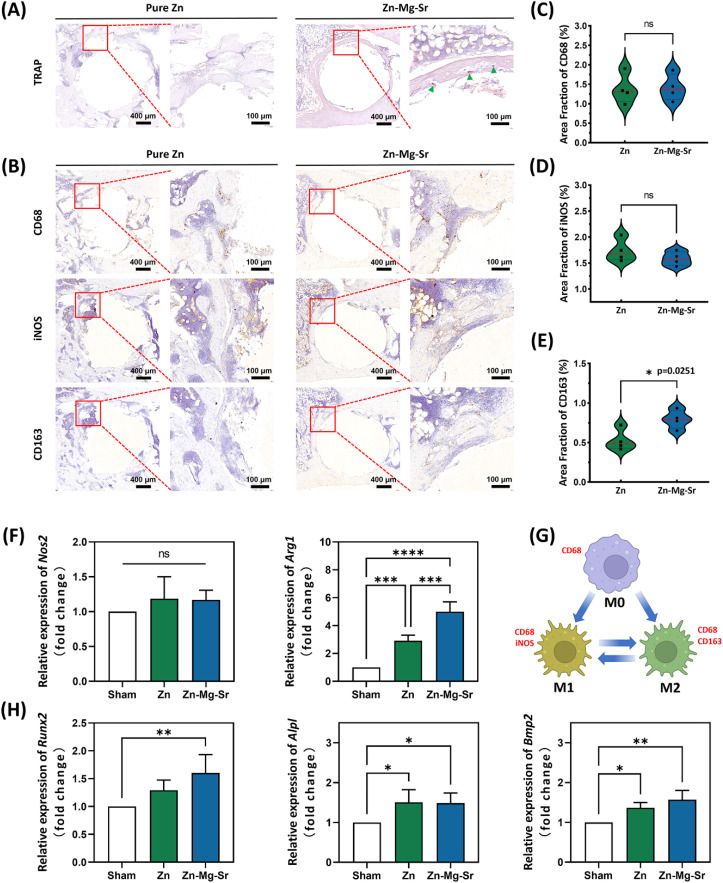
The effects of Zn and Zn-0.8Mg-0.2Sr on osteoclast activity, macrophage polarization, and osteogenic gene expression. (A) Tartrate-resistant acid phosphatase (TRAP) staining images. Green arrows indicate the presence of osteoclasts; (B) immunohistochemical staining of CD68, iNOS, and CD163 (*n* = 4); (C)–(E) present the semi-quantitative analyses of area fraction of CD68, iNOS, and CD163, respectively; (F) relative expression levels of *Nos2* and *Arg1* genes related to macrophage polarization (*n* = 4); (G) schematic illustration of the dynamic changes among M0, M1, and M2 macrophages and their corresponding markers in this study; (H) relative expression levels of *Runx2*, *Alpl*, and *Bmp2* genes related to osteogenesis (*n* = 4). Asterisks indicate statistically significant differences (*p < 0.05, **p < 0.01, ***p < 0.001, and ****p < 0.0001).

Zn has been reported to inhibit osteoclastogenesis in a dose-dependent manner.^60^ For instance, a recent study demonstrated that a Zn-0.8Mg alloy exhibited bidirectional regulatory properties, simultaneously promoting osteoblast differentiation and suppressing osteoclast differentiation.^61^ However, in the present study, TRAP staining (Fig. 7A) displayed the scarcity of osteoclasts surrounding pure Zn, while the presence of osteoclasts (indicated by green arrow) around Zn-0.8Mg-0.2Sr. This may be attributed to Zn^2+^ level released from Zn-0.8Mg-0.2Sr falling below the threshold required to inhibit osteoclast differentiation. Moreover, previous research has indicated that the presence of Sr may interfere with the inhibitory effect of Zn on osteoclasts.^62^ It should be noted that inhibiting osteoclast differentiation is not necessarily beneficial for osteogenesis, as osteoclasts play a crucial role in maintaining bone homeostasis.^63,64^ During bone repair, osteoclasts are essential for resorbing old or damaged bone, thereby facilitating the renewal and remodeling of bone tissue. Conversely, excessive inhibition of osteoclast activity may impede osteogenesis.^65^

Once an implant is introduced into the body, it immediately interacts with the immune system.^66^ The immune response to the implant is crucial in determining the outcome of bone healing.^67^ Macrophages, as key mediators of the immune system, play a central role in the bone healing process by activating and differentiating into pro-inflammatory M1 or anti-inflammatory M2 phenotypes.^68,69^ In the early stages of bone repair, M1 macrophages secrete pro-inflammatory cytokines such as IL-1, IL-6, and TNF-α to eliminate pathogens and debris, creating a microenvironment that facilitates bone repair. However, prolonged pro-inflammatory responses may result in chronic inflammation and delayed healing, emphasizing the importance of timely transition to the M2 phenotype to promote bone healing.^70,71^ In this study, immunohistochemical analysis (Fig. 7B–E) revealed no significant differences in the number of CD68-positive cells (pan-macrophages) and iNOS-positive cells (M1 macrophages) between the pure Zn and Zn-0.8Mg-0.2Sr groups. However, the number of CD163-positive cells (M2 macrophages) surrounding Zn-0.8Mg-0.2Sr implant was significantly higher than that around pure Zn. The increased M2 macrophages might derive from M1-to-M2 transition or direct activation of M0 macrophages. This finding suggests that Zn-0.8Mg-0.2Sr implants exhibit superior immunomodulatory properties, promoting a favorable immune microenvironment conducive to bone healing and tissue regeneration. In addition to Zn, both Mg and Sr have been reported to promote the polarization of M2 macrophages.^72–75^ Presumably, the synergistic effects of Zn^2+^, Mg^2+^, and Sr^2+^ could enhance the immunomodulatory capacity of Zn-0.8Mg-0.2Sr alloy by facilitating a more balanced inflammatory response.

To further evaluate the *in vivo* effects of pure Zn and Zn-0.8Mg-0.2Sron macrophage polarization and osteogenic differentiation, the relative expression levels of *Nos2*, *Arg1*, *Runx2*, *Alpl*, and *Bmp2* were assessed using qRT-PCR. Consistent with the immunohistochemical findings, no significant differences were found in the expression of M1 macrophage marker gene (*Nos2*) among the sham control, pure Zn, and Zn-0.8Mg-0.2Sr groups. However, the expression of M2 macrophage marker gene (*Arg1*) was notably upregulated in the pure Zn and Zn-0.8Mg-0.2Sr groups compared to the sham control (Fig. 7F). For clarity, the dynamic transitions among M0, M1, and M2 macrophages and their corresponding markers are illustrated in Fig. 7G. Osteogenesis-related gene expression (Fig. 7H) exhibited a similar trend to the osteogenic phenotype results (Fig. 6D) but was not entirely consistent. Specifically, compared to the sham group, expression levels of *Runx2*, *Alpl*, and *Bmp2* were upregulated in both the Zn and Zn-0.8Mg-0.2Sr groups. However, no significant difference was found between the Zn and Zn-0.8Mg-0.2Sr groups, although the mean expression levels of *Runx2* and *Bmp2* in the Zn-0.8Mg-0.2Sr group were slightly higher. This may be due to a limitation of the study: to obtain sufficient RNA for detection, a larger volume of bone tissue was required, including not only the bone tissue surrounding the implant but also some distal bone. This may reduce the resolution of group differences in gene expression. To address this limitation, microdissection techniques and single-cell RNA sequencing could be employed in future studies to precisely localize gene expression around implants, thus providing a deeper understanding of ion interactions and biological mechanisms.

## 4. Conclusion

The biodegradable Zn-0.8Mg-0.2Sr ternary alloy exhibits controlled and uniform degradation kinetics mediated by a dense, phosphorus-rich passivation layer, mitigating pitting corrosion and structural collapse. Within the non-cytotoxic threshold (Zn^2+^ ≤8.98 μg mL^−1^), Zn-0.8Mg-0.2Sr extracts effectively enhance osteogenesis, driven by synergistic effects of Zn^2+^, Mg^2+^, and Sr^2+^. Importantly, Zn-0.8Mg-0.2Sr demonstrates favorable *in vivo* biocompatibility and biofunctionality, promoting anti-inflammatory immunomodulation, bone regeneration, and bone remodeling. These findings highlight Zn-0.8Mg-0.2Sr as a promising alternative to conventional internal fixation materials for orthopedic applications.

## Ethics approval

The animal study was approved by the Ethics Committee of West China Hospital of Stomatology (No. WCHSIRB-D-2020-006) and was conducted at the Animal Experiment Center of West China Hospital with animal welfare followed in accordance with international guidelines.

## Author contributions

Yuting Tian: data curation, formal analysis, investigation, methodology, visualization, writing – original draft, writing review & editing. Yichen Xu: conceptualization, data curation, formal analysis, funding acquisition, methodology, visualization, writing – original draft, writing – review & editing. Pinc Jan: investigation, methodology, writing – review & editing. Jaroslav Fojt: investigation, methodology, writing – review & editing. Vojtěch Hybášek: methodology, writing – original draft, writing – review& editing. Jiří Kubásek: data curation, investigation, writing – review & editing. Šárka Msallamová: investigation, writing – review & editing. Yong Xiang: investigation, writing – review & editing. Min Guo: funding acquisition, supervision. Jaroslav Čapek: funding acquisition, methodology, supervision, writing – original draft, writing – review & editing. Ping Li: conceptualization, funding acquisition, project administration, supervision, validation, writing – review & editing. Tao Hu: conceptualization, funding acquisition, project administration, resources, supervision, validation, writing – review & editing.

## Conflicts of interest

There are no conflicts to declare.

## Supplementary Material

RA-015-D5RA02009C-s001

## Data Availability

All data supporting the findings of this study are available within the Manuscript and its SI. No additional datasets were generated or analyzed during this study. Oligonucleotide primers used in qRT-PCR (Table S1); pictures of Zn-0.8Mg-0.2Sr and pure Zn after 72 hours of *in vitro* corrosion test (Fig. S1); scanning electron microscopy images and energy dispersive spectroscopy analysis sites of Zn-0.8Mg-0.2Sr, Zn-1Mg and pure Zn (Fig. S2–S4); the atomic percentage of the various elements in each spectrum of Zn-0.8Mg-0.2Sr, Zn-1Mg and pure Zn (Tables S2–S4); comparison of polarization and OCP curves for Zn-0.8Mg-0.2Sr, Zn-1Mg and pure Zn (Fig. S5). Comparison of Nyquist curves for Zn-0.8Mg-0.2Sr, Zn-1Mg and pure Zn (Fig. S6); evolution of equivalent circuit elements, capacitances from the CPE element were calculated according to Brug *et al.*, (G.J. Brug, A. G. van Eeden, M. Sluytera-Rehbach, J. H Sluyters, The Analysis of Electrode Impedances Complicated by the Presence of a Constant Phase Element, Journal of Electroanalytic Chemistry, 176 (1984) 275–295) (Fig. S7). Thermodynamic simulation of stable corrosion products using hydra/medusa software (Fig. S8). The contents (μg mL^−1^) of Zn^2+^, Mg^2+^, and Sr^2+^ in blank medium (Table S5). The contents (μg mL^−1^) of Zn^2+^, Mg^2+^, and Sr^2+^ in different pure Zn extracts dilutions and Zn-0.8Mg-0.2Sr extracts dilutions (Tables S6 and S7). See DOI: https://doi.org/10.1039/d5ra02009c.
