# Supplementary material for: Biodegradable Zn-0.8Mg-0.2Sr alloy as an internal fixation material exhibits controlled degradation with enhanced osteogenesis
Source: RSC Adv. 2025 Aug 22;15(37):30071–88. doi: 10.1039/d5ra02009c (PMC12376982; doi:10.1039/d5ra02009c)
Supplement: RA-015-D5RA02009C-s001 [file RA-015-D5RA02009C-s001.pdf]

Supplementary Information (SI) for RSC Advances.
This journal is © The Royal Society of Chemistry 2025

Supplementary Information

Biodegradable Zn-0.8Mg-0.2Sr Alloy as an Internal Fixation Material Exhibits Controlled Degradation with Enhanced Osteogenesis

Yuting Tian, [*a] Yichen Xu, [*a] Jan Pinc, [b] Jaroslav Fojt, [c] Vojtěch Hybášek, [c] Jiří Kubásek, [c] Šárka Msallamová, [c] Yong Xiang, [a] Min Guo, [d] Jaroslav Čapek, [#b] Ping Li, [#e] Tao Hu [#a]

[a] State Key Laboratory of Oral Diseases & National Center for Stomatology & National Clinical Research Center for Oral Diseases & Frontier Innovation Center for Dental Medicine Plus, West China Hospital of Stomatology, Sichuan University, Chengdu 610041, China

[b] FZU – The Institute of Physics, Czech Academy of Sciences, Na Slovance 1999/2, Prague 8 182 00, Czech Republic

[c] University of Chemistry and Technology Prague, Faculty of Chemical Technology, Department of Metals and Corrosion Engineering, Prague 6 – Dejvice, Czech Republic

[d] SINOPEC Key Laboratory of Research and Application of Medical and Hygienic Materials, SINOPEC Beijing Research Institute of Chemical Industry Co., Ltd., Beijing, 100013, China

[e] Department of Prosthodontics, School and Hospital of Stomatology & Guangdong Engineering Research Center of Oral Restoration and Reconstruction & Guangzhou Key Laboratory of Basic and Applied Research of Oral Regenerative Medicine, Guangzhou Medical University, Guangzhou 510180, China

***These authors contributed equally as co-first authors to this work.**

[#]Corresponding authors:

PhD. Jaroslav Čapek

Email: capekj@fzu.cz

Prof. Dr. Ping Li

Email: pingli@gzhmu.edu.cn

Prof. Dr. Tao Hu

Email: hutao@scu.edu.cn

Table S1. Oligonucleotide primers used in qRT-PCR.

Gene	ID	Forward Primer	Reverse Pimer
Gapdh	24383	ACGGCAAGTTCAACGGCACAG	CGACATACTCAGCACCAGCATCAC
Nos2	24599	TCTTGGAGCGAGTTGTGGATTGTTC	AGTGATGTCCAGGAAGTAGGTGAGG
Arg1	29221	AGTGTGGTGCTGGGTGGAGAC	GCGGAGTGTTGATGTCAGTGTGAG
Runx2	367218	CTTCGTCAGCGTCCTATCAGTTCC	TCCATCAGCGTCAACACCATCATTC
Alpl	25586	CACGGCGTCCATGAGCAGAAC	CAGGCACAGTGGTCAAGGTTGG

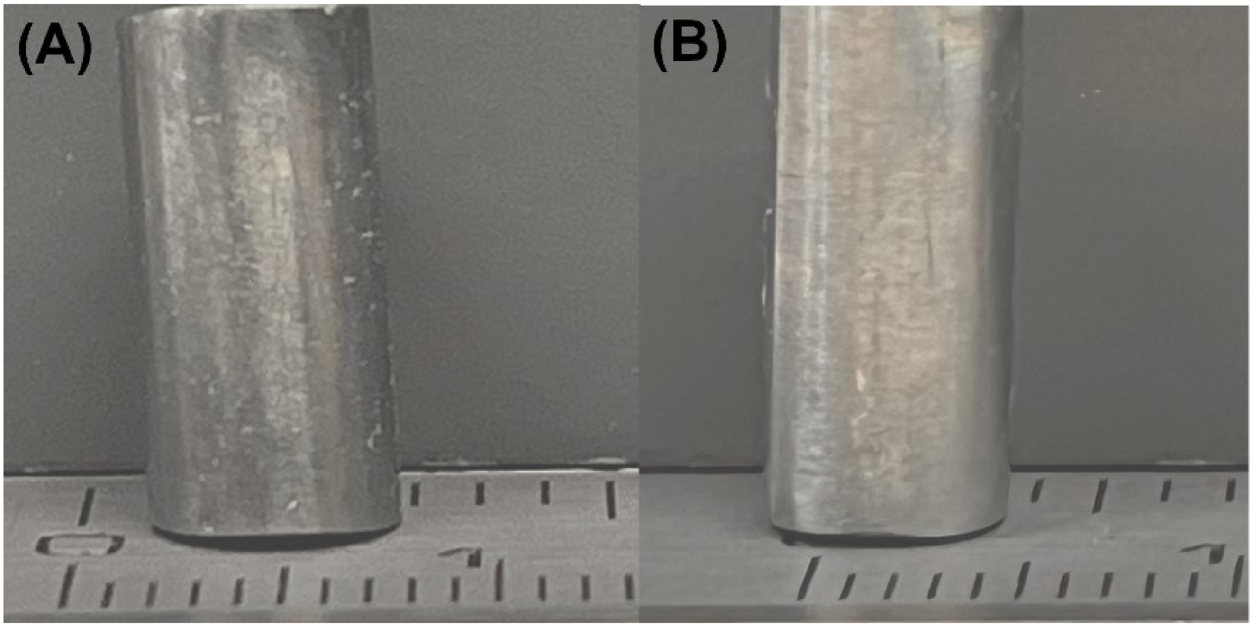

Figure S1. Pictures of (A) Zn-0.8Mg-0.2Sr and (B) pure Zn after 72 hours of *in vitro* corrosion test.

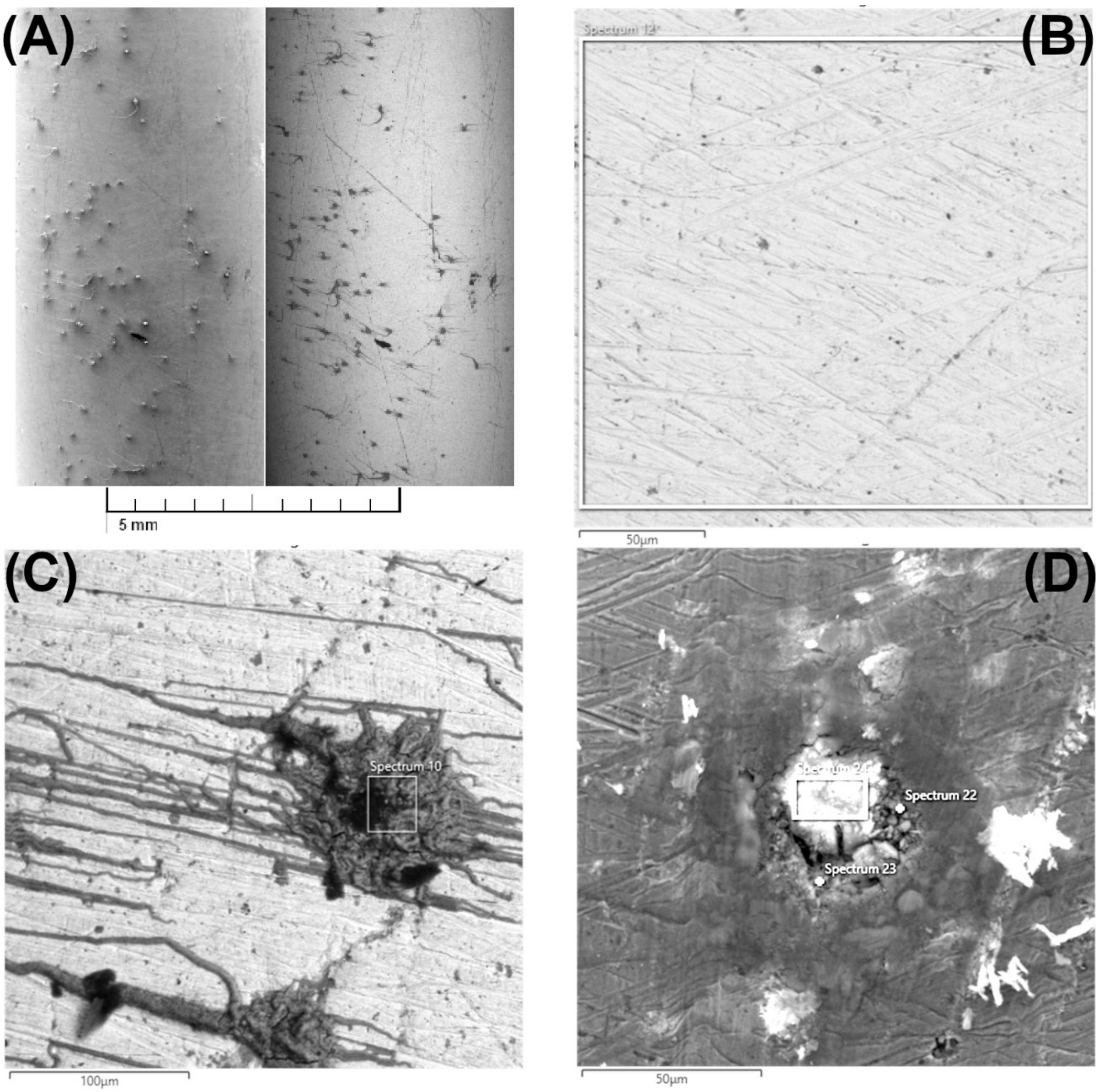

Figure S2. Scanning electron microscopy images and energy dispersive spectroscopy analysis sites of Zn-0.8Mg-0.2Sr after 72h of exposure;(A) overall view; (B) unattacked surface; (C) fibrous deposit; (D) after its mechanical removal.

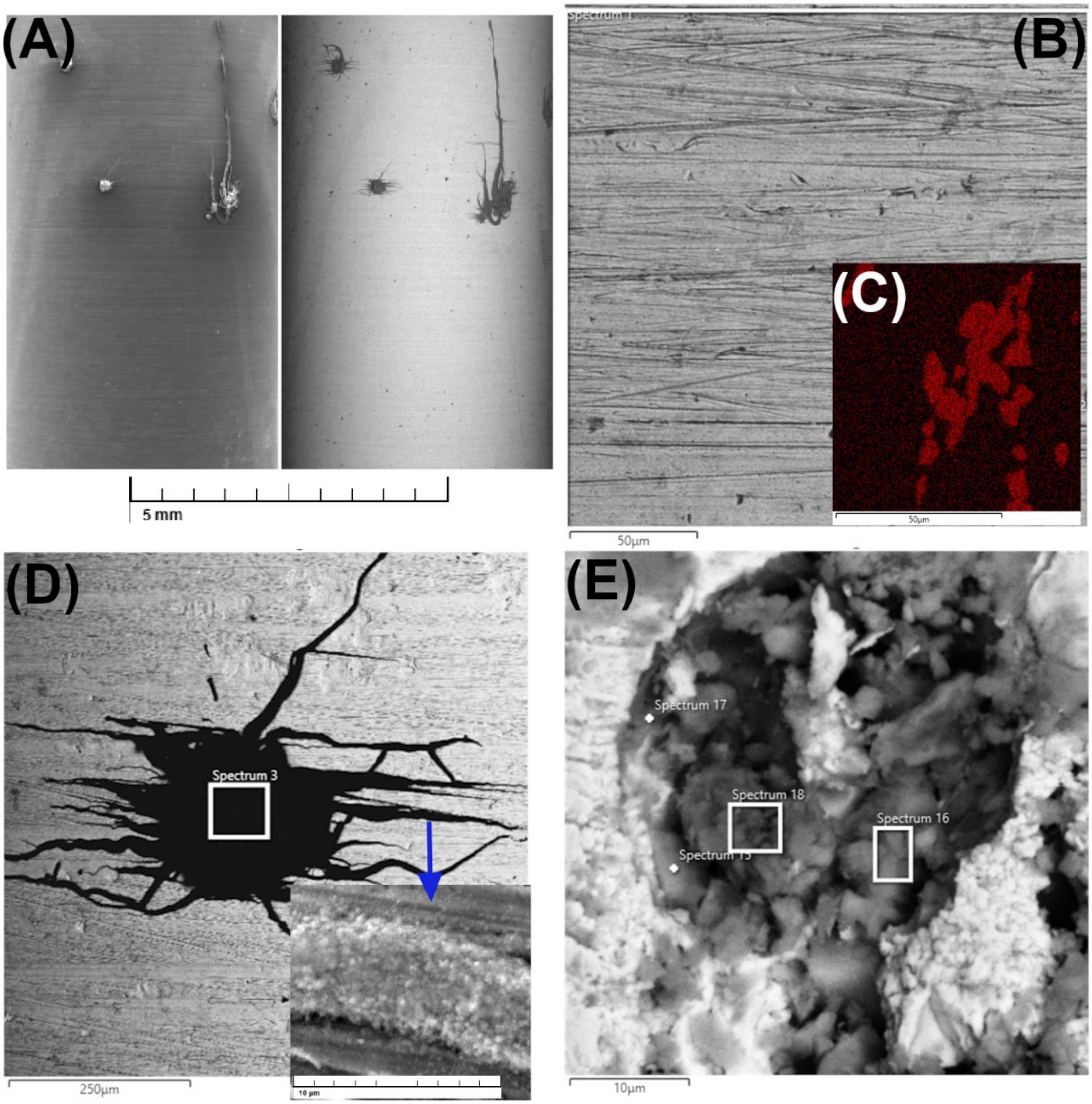

Figure S3. Scanning electron microscopy images and energy dispersive spectroscopy analysis sites of Zn-1Mg;(A) overall view after exposure; (B) Mg distribution showing the size of the Mg2Zn11 phase (C) unattacked surface after exposure; (C) surface after exposure with fibrous deposit; (D) after its mechanical removal.

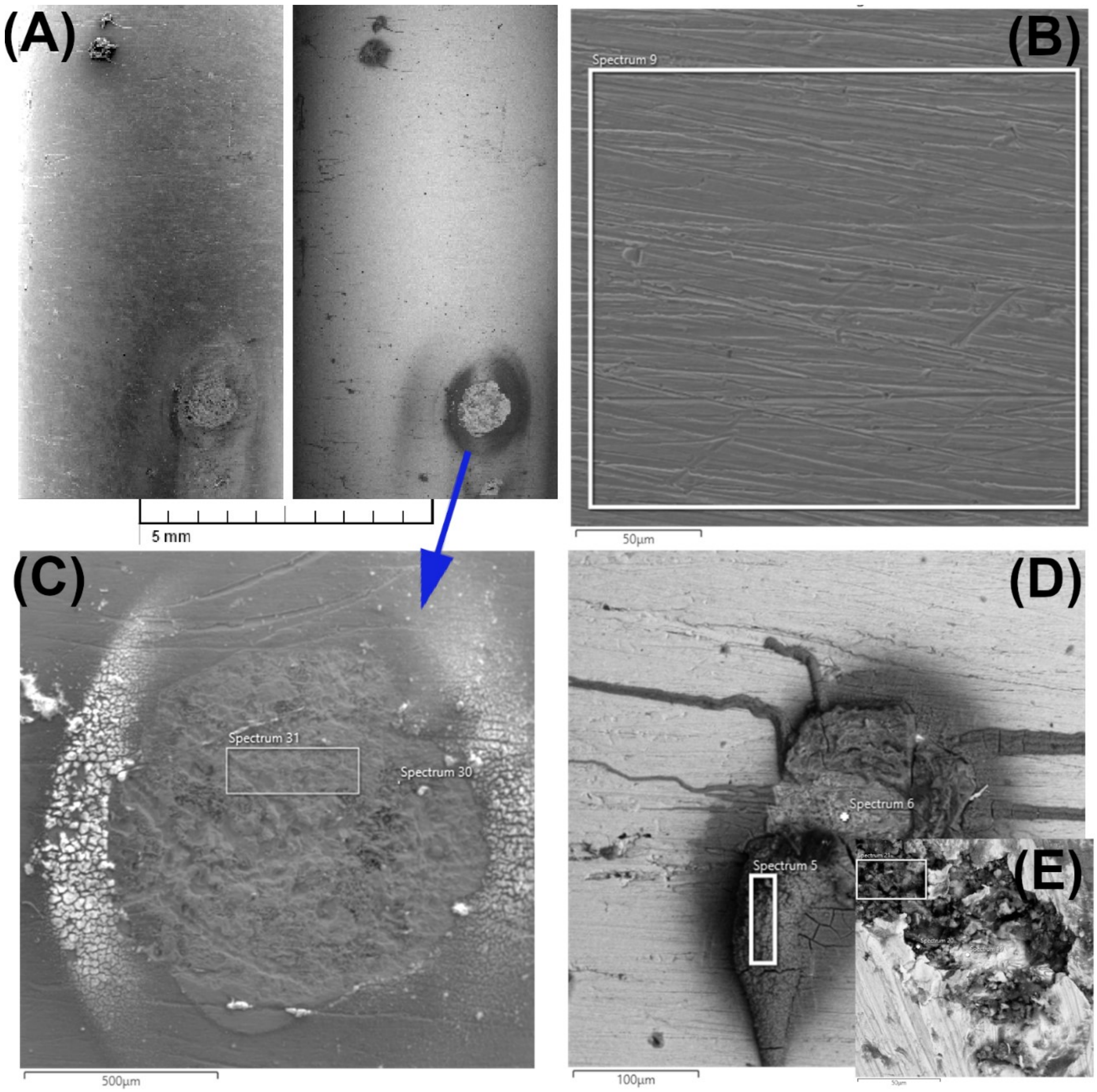

Figure S4. Scanning electron microscopy images and energy dispersive spectroscopy analysis sites of pure Zn after exposure; (A) overall view; (B) unattacked surface; (C) attacked surface; (D) fibrous deposit; (E) after its mechanical removal.

Table S2. The atomic percentage of the various elements in each spectrum of Zn-0.8Mg-0.2Sr.

	Spectrum 12	Spectrum 10	Spectrum 22	Spectrum 23	Spectrum 24
**description**	**bare surface**	**fibrous deposit**	**under deposit**		
**C***	37.2	57.1	30.9	27.9	38.5
**O***	11.6	31.5	40.5	29.9	38.4
**Mg**	2.1	0.3	0.5	0.4	0.5
**P**	0.3	2.1	4.4	3.8	4.0
**S**		0.4	0.5	0.3	0.5
**Cl**		0.7	2.1	2.4	2.2
**K**		0.2	0.1		
**Ca**	0.1	0.8	1.0	0.6	0.4
**Zn**	48.8	7.0	20.2	34.7	15.6
**Sr**					0.0
**P/Zn**	0.006	0.3	0.2	0.1	0.3
**O/Zn**	0.2	4.5	2.0	0.9	2.5
**Mg/Zn**	0.04	0.04	0.03	0.01	0.03

* – the analysis of these elements is only indicative

Table S3. The atomic percentage of the various elements in each spectrum of Zn-1Mg.

	Spectrum 1	Spectrum 3	Spectrum 18	Spectrum 15	Spectrum 16	Spectrum 17
**description**	**bare surface**	**fibrous deposit**	**under deposit**			
**C***	44.1	36.1	24.7	19.8	29.5	36.8
**O***	5.5	47.8	36.0	10.2	27.0	25.5
**Mg**	0.9	0.5	0.8			
**P**		3.6	9.6	0.7	1.1	1.2
**S**		0.3	0.4	0.1	0.4	0.3
**Cl**		0.6	1.0	1.0	4.7	1.7
**K**		0.1	0.1			
**Ca**		1.4	6.0	0.1		0.2
**Zn**	49.6	9.6	21.4	68.0	37.2	34.4
**P/Zn**		0.4	0.4	0.01	0.03	0.04
**O/Zn**	0.1	5.0	1.7	0.2	0.7	0.7
**Mg/Zn**	0.02	0.05	0.04			

* – the analysis of these elements is only indicative

Table S4. The atomic percentage of the various elements in each spectrum of pure Zn.

	Spectrum 9	Spectrum 31	Spectrum 30	Spectrum 5	Spectrum 6	Spectrum 19	Spectrum 20	Spectrum 21
**description**	**bare surface**	**attacked surface**		**fibrous deposit**		**under deposit**		
**C**	37.0	46.6	34.0	57.4	41.1	16.4	30.6	30.52
**O**	5.9	9.5	3.4	33.3	26.9	17.2	35.5	31.54
**Mg**		0.06	0.17		0.13			
**P**	0.2	0.3		3.4		0.3	3.8	1.29
**S**		0.1		0.3	0.3	0.1	0.5	0.5
**Cl**		0.1	0.1	0.2	5.8	1.3	0.9	3.51
**K**				0.2			0.1	
**Ca**	0.1	0.1		1.7		0.1	1.4	0.35
**Zn**	56.8	43.3	62.4	3.6	25.8	64.7	27.3	32.29
**P/Zn**	0.004	0.007		1		0.005	0.1	0.04
**O/Zn**	0.1	0.2	0.1	9.3	1	0.3	1.3	1.0

* – analysis of the analysis of these elements is only indicative

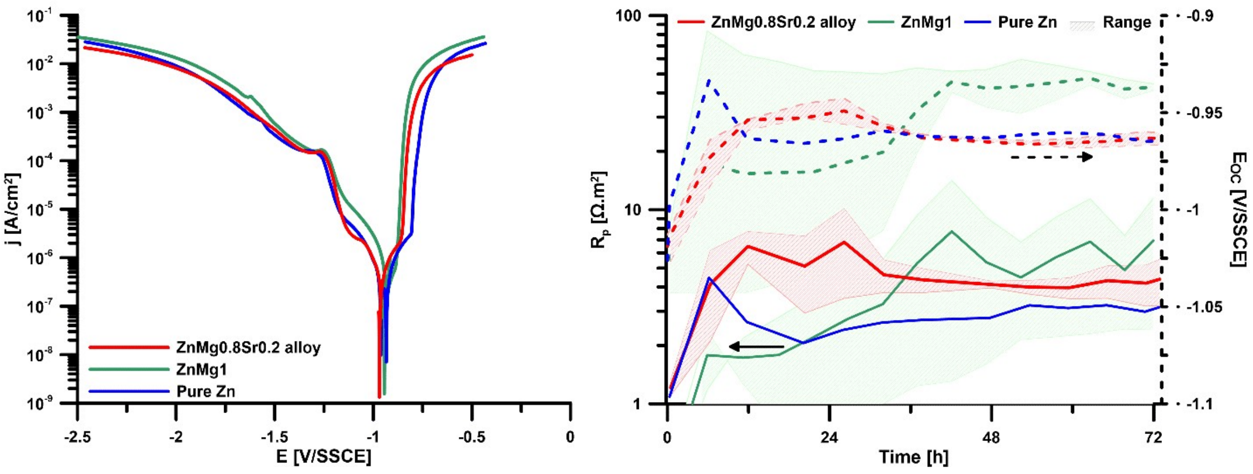

Figure S5. Comparison of polarization and OCP curves for Zn-0.8Mg-0.2Sr, Zn-1Mg and pure Zn.

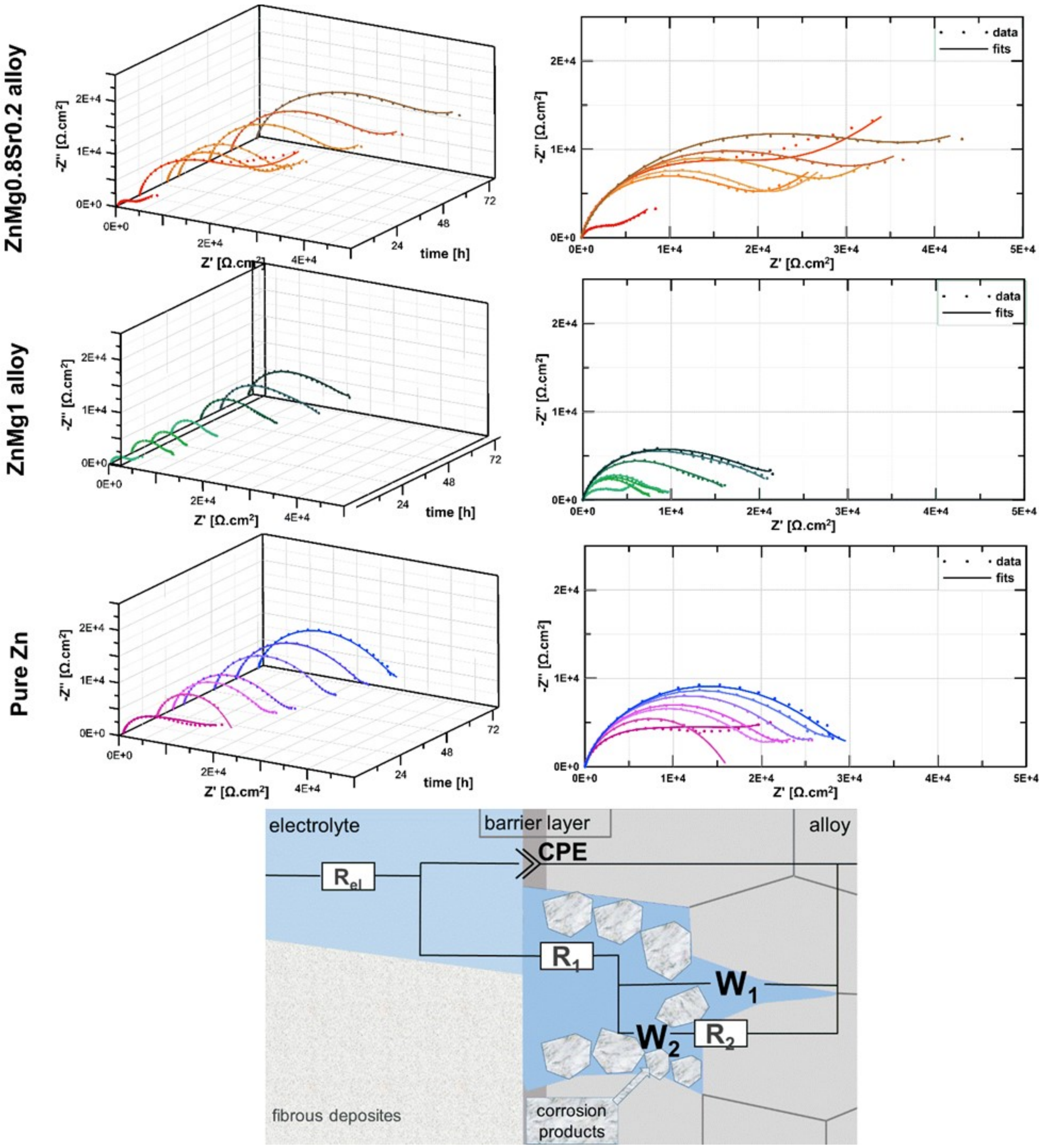

Figure S6. Comparison of Nyquist curves for Zn-0.8Mg-0.2Sr, Zn-1Mg and pure Zn.

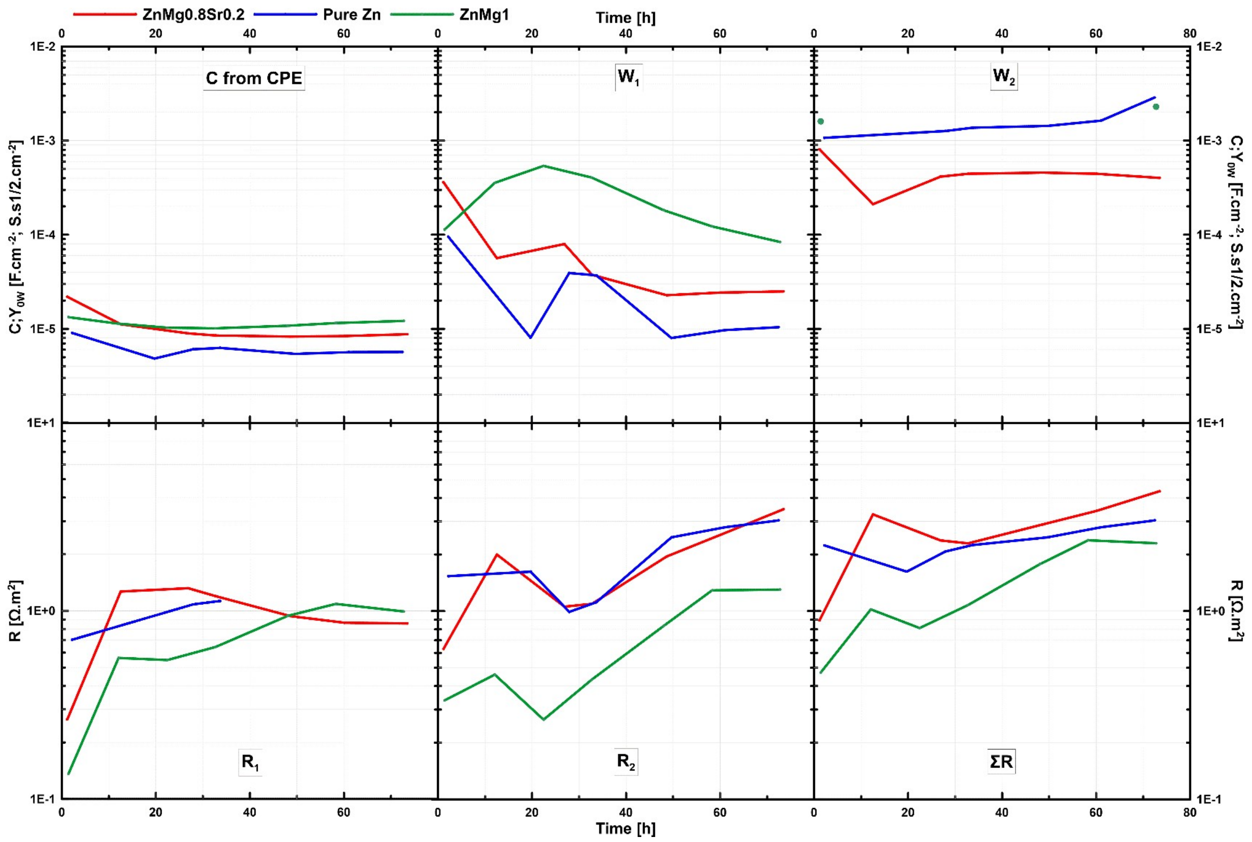

Figure S7. Evolution of equivalent circuit elements, capacitances from the CPE element were calculated according to Brug et al., (G.J. Brug, A. G. van Eeden, M. Sluytera-Rehbach, J. H Sluyters, The Analysis of Electrode Impedances Complicated by the Presence of a Constant Phase Element, Journal of Electroanalytic Chemistry, 176 (1984) 275-295).

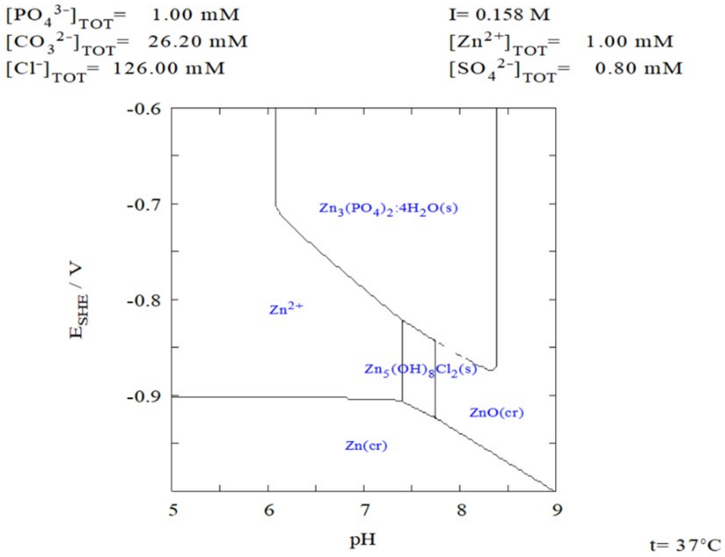
[PO4 3-]TOT= 1.00 mM
[CO3 2-]TOT= 26.20 mM
[Cl-]TOT= 126.00 mM
I= 0.158 M
[Zn2+]TOT= 1.00 mM
[SO4 2-]TOT= 0.80 mM
Zn3(PO4)2:4H2O(s)
Zn2+
Zn5(OH)8Cl2(s)
ZnO(cr)
Zn(cr)
ESHE / V
pH
t= 37°C

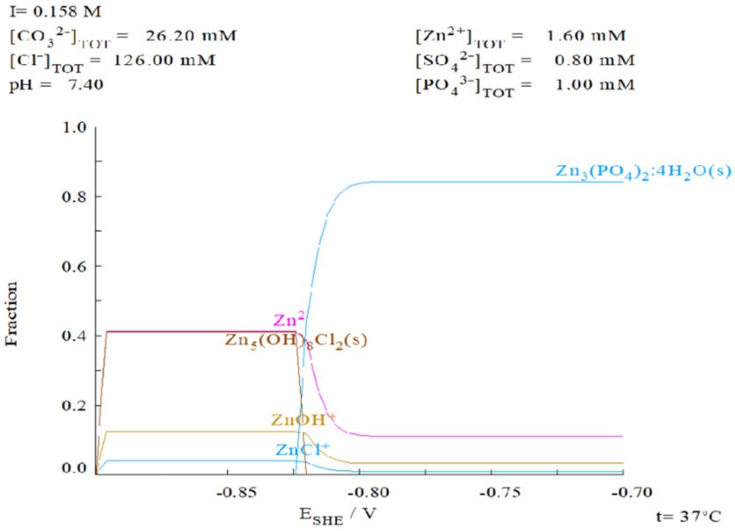
I= 0.158 M
[CO3 2-]TOT = 26.20 mM
[Cl-]TOT = 126.00 mM
pH = 7.40
[Zn2+]TOT = 1.60 mM
[SO4 2-]TOT = 0.80 mM
[PO4 3-]TOT = 1.00 mM
Zn3(PO4)2:4H2O(s)
Zn2
Zn5(OH)8Cl2(s)
ZnOH+
ZnCl+
Fraction
ESHE / V
t= 37°C

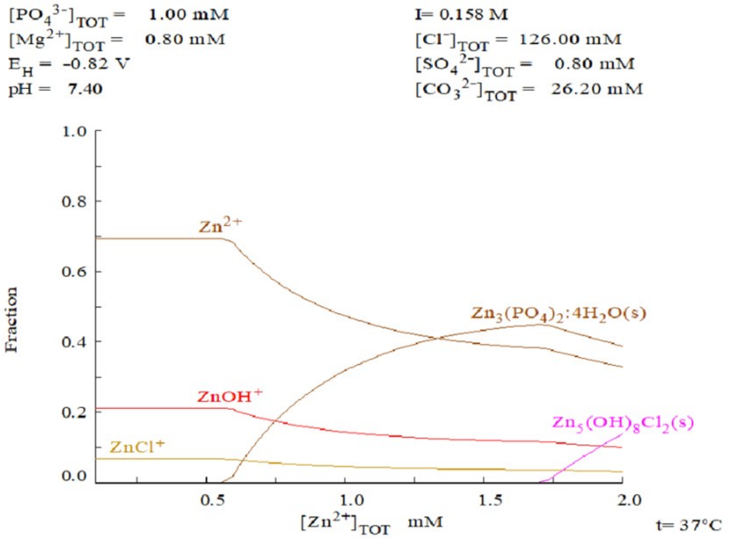
[PO4 3-]TOT = 1.00 mM
[Mg2+]TOT = 0.80 mM
EH = -0.82 V
pH = 7.40
I= 0.158 M
[Cl-]TOT = 126.00 mM
[SO4 2-]TOT = 0.80 mM
[CO3 2-]TOT = 26.20 mM
Zn2+
Zn3(PO4)2:4H2O(s)
ZnOH+
Zn5(OH)8Cl2(s)
ZnCl+
Fraction
[Zn2+]TOT mM
t= 37°C

Figure S8: Thermodynamic simulation of stable corrosion products using hydra/medusa software

Table S5. The contents (μg/mL) of Zn^{2+}, Mg^{2+}, and Sr^{2+} in blank medium.

	**Zn^{2+}**	**Mg^{2+}**	**Sr^{2+}**
**Original**	0.24	20.97	0.00

Table S6. The contents (μg/mL) of Zn^{2+}, Mg^{2+}, and Sr^{2+} in different pure Zn extracts dilutions (diluted by blank medium).

	**Zn^{2+}**	**Mg^{2+}**	**Sr^{2+}**
**Original**	97.03	20.23	0.00
**Normalized (100%)**	35.20	20.70	0.00
**50%**	17.72	20.84	0.00
**25%**	8.98	20.90	0.00
**10%**	3.74	20.94	0.00

Table S7. The contents (μg/mL) of Zn^{2+}, Mg^{2+}, and Sr^{2+} in different Zn-0.8Mg-0.2Sr extracts dilutions (diluted by blank medium).

	**Zn^{2+}**	**Mg^{2+}**	**Sr^{2+}**
**Original**	35.20	22.73	0.15
**Normalized (100%)**	35.20	22.73	0.15
**50%**	17.72	21.85	0.08
**25%**	8.98	21.41	0.04
**10%**	3.74	21.15	0.02
